# Supplementary material for: 3-Aryl-5-aminobiphenyl Substituted [1,2,4]triazolo[4,3-c]quinazolines: Synthesis and Photophysical Properties
Source: Molecules. 2023 Feb 17;28(4):1937. doi: 10.3390/molecules28041937 (PMC9963873; doi:10.3390/molecules28041937)
Supplement: Supplementary file 1 [file molecules-28-01937-s001.zip › molecules-2208975-supplementary.pdf]

3-Aryl-5-aminobiphenyl substituted [1,2,4]triazolo[4,3-*c*]quinazolines: synthesis and photophysical properties

Alexandra E. Kopotilova, Tatyana N. Moshkina, Emiliya V. Nosova, Galina N. Lipunova, Ekaterina S. Starnovskaya, Dmitry S. Kopchuk, Grigory A. Kim, Vasiliy S. Gaviko, Pavel A. Slepukhin and Valery N. Charushin

CONTENTS

1. NMR and mass spectra of 3-aryl-5-aminobiphenyl substituted [1,2,4]triazolo[4,3-*c*]quinazolines 2
2. Crystallographic data of compounds **2a** and **2e** 21
3. Absorption and emission spectra of fluorophores in toluene and MeCN 26
4. Emission spectra of fluorophores in solid state 39
5. Solvatochromic study for compounds **2** 40
6. Absorption and emission behavior of compounds **2** in MeCN/water mixture 43
7. Absorption and fluorescence behavior of compounds **2e** and **2h** in acidic media 48

1. NMR and mass spectra of 3-aryl-5-aminobiphenyl substituted [1,2,4]triazolo[4,3-*c*]quinazolines

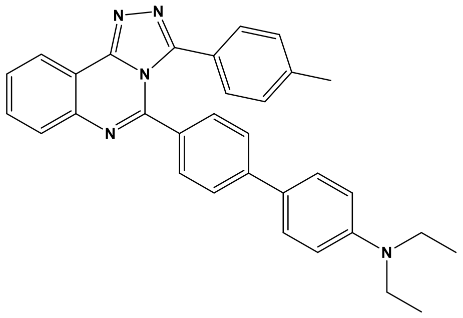

2a

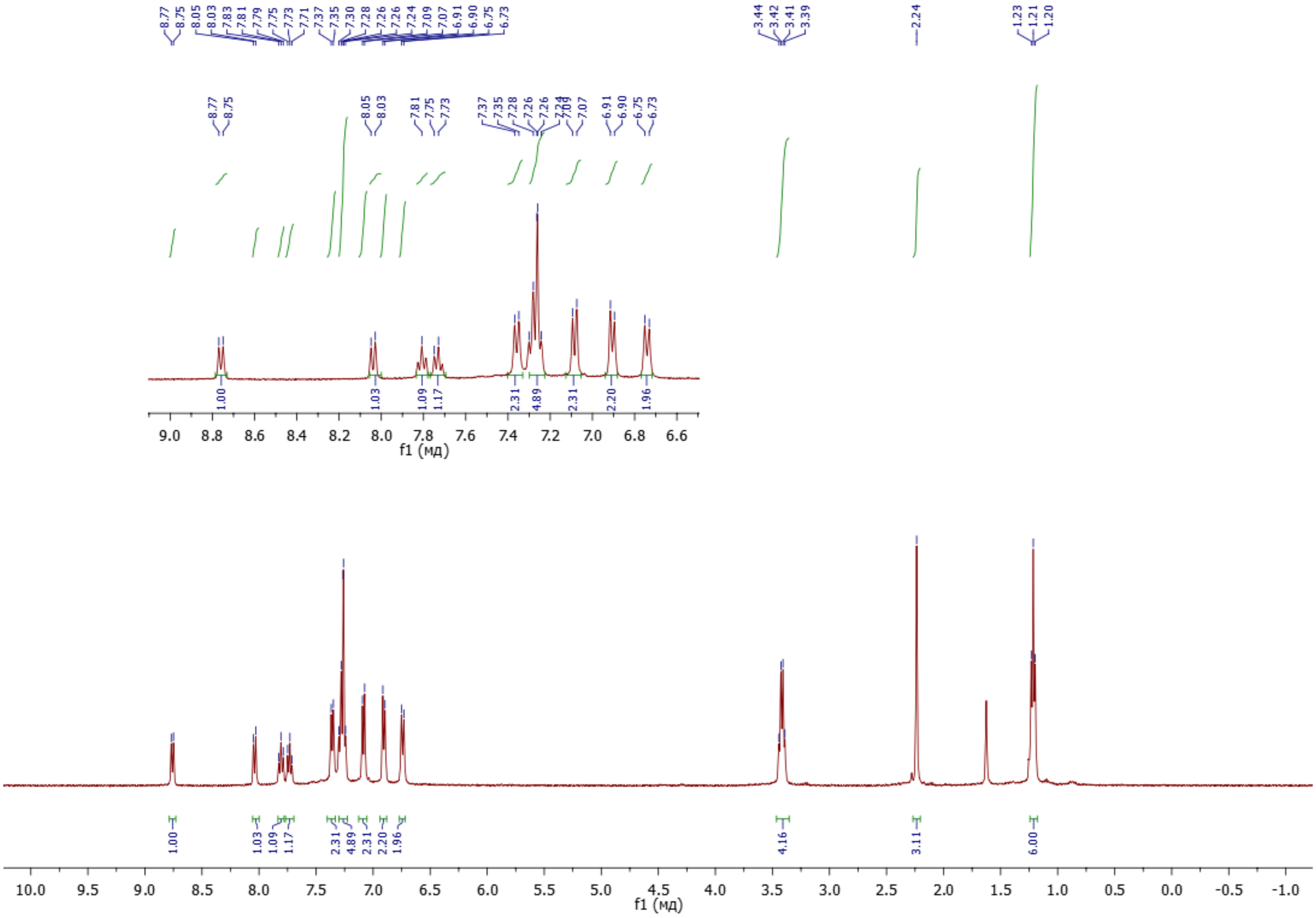

a

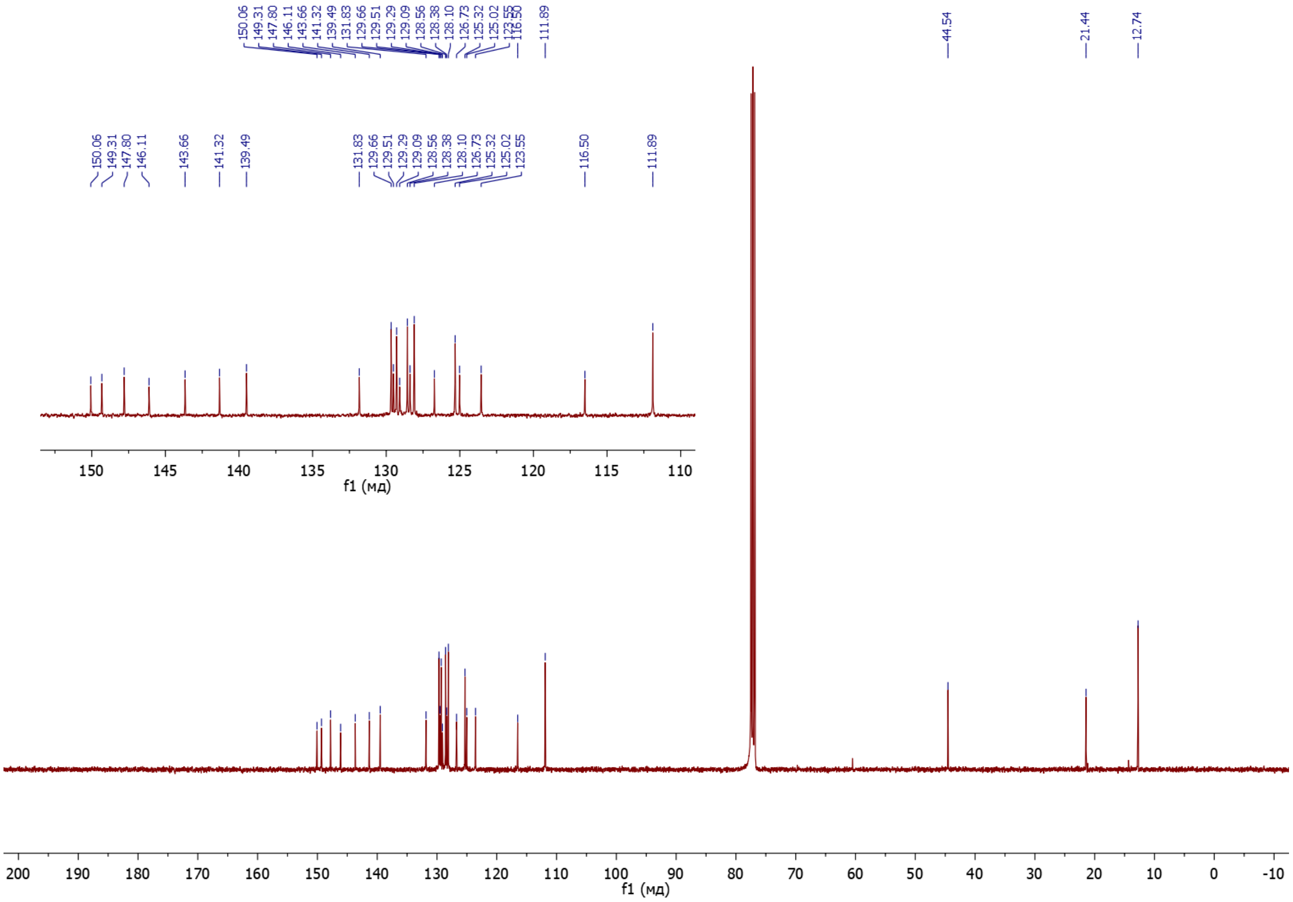

b

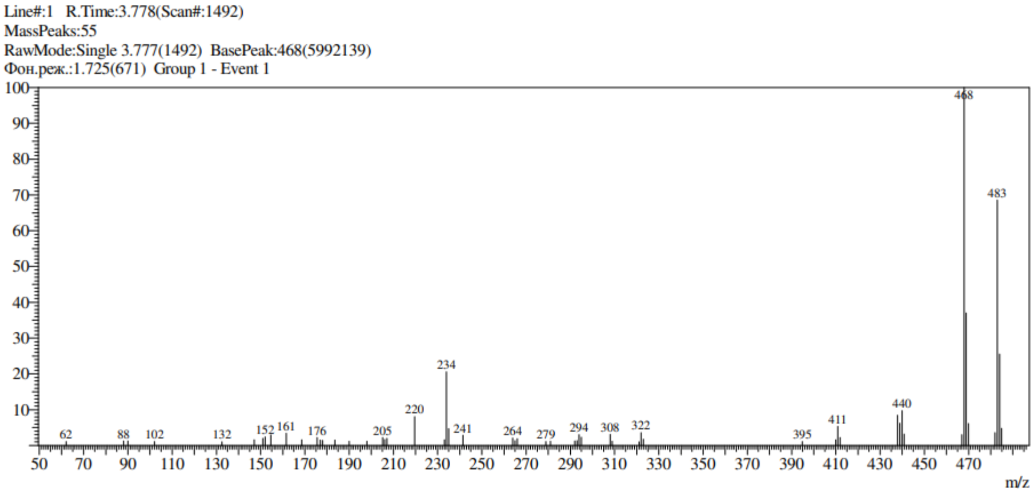

c

Figure S1. ^{1}H NMR spectrum (a) and ^{13}C NMR spectrum in $CDCl_3$ (b); mass spectrum (c) of **2a**.

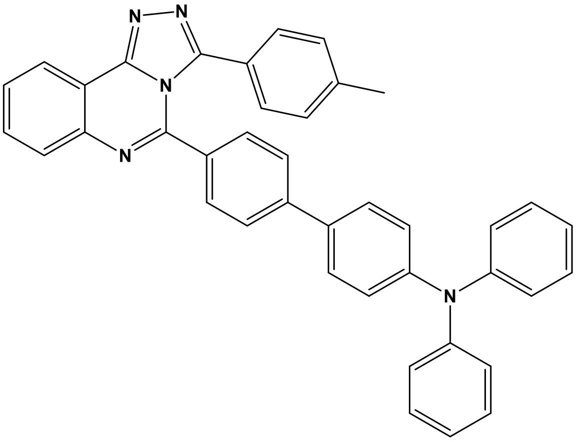

2b

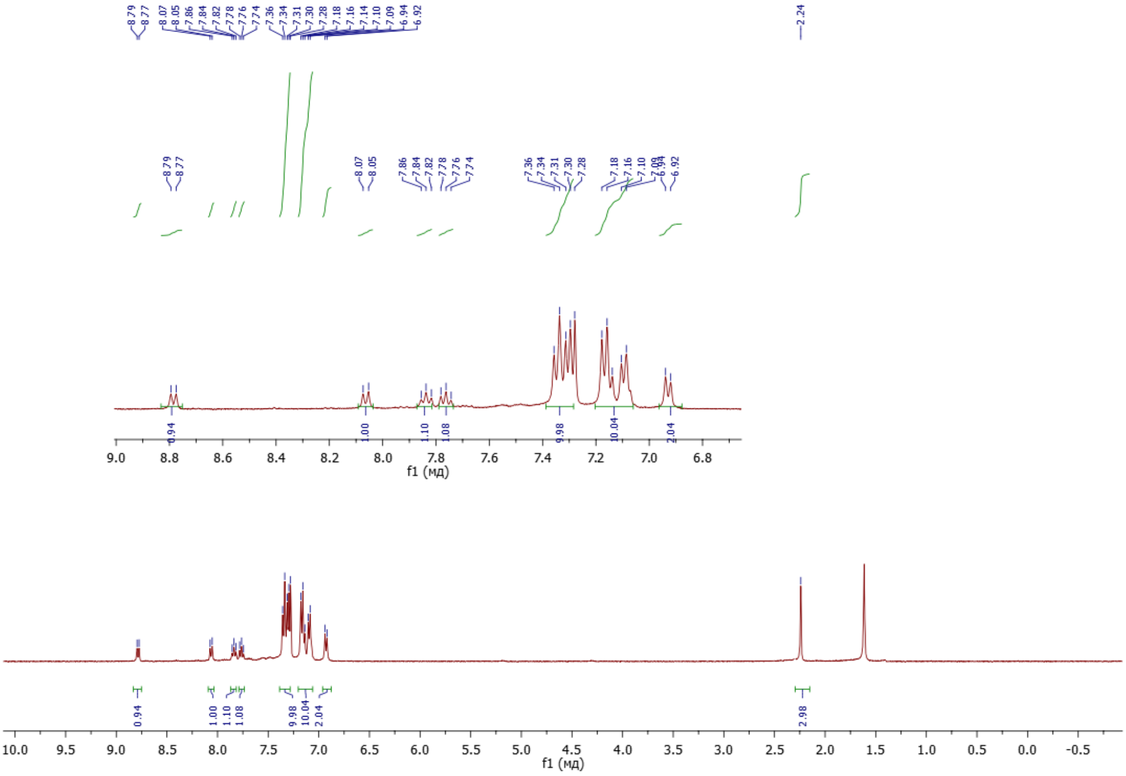
8.79 8.77 8.07 8.05 7.86 7.84 7.82 7.78 7.76 7.74 7.36 7.34 7.31 7.30 7.28 7.18 7.16 7.14 7.10 7.09 6.94 6.92
2.24
0.94 1.00 1.10 1.08 9.98 10.04 2.04
9.0 8.8 8.6 8.4 8.2 8.0 7.8 7.6 7.4 7.2 7.0 6.8
f1 (мд)
0.94 1.00 1.10 1.08 9.98 10.04 2.04 2.98
10.0 9.5 9.0 8.5 8.0 7.5 7.0 6.5 6.0 5.5 5.0 4.5 4.0 3.5 3.0 2.5 2.0 1.5 1.0 0.5 0.0 -0.5
f1 (мд)

a

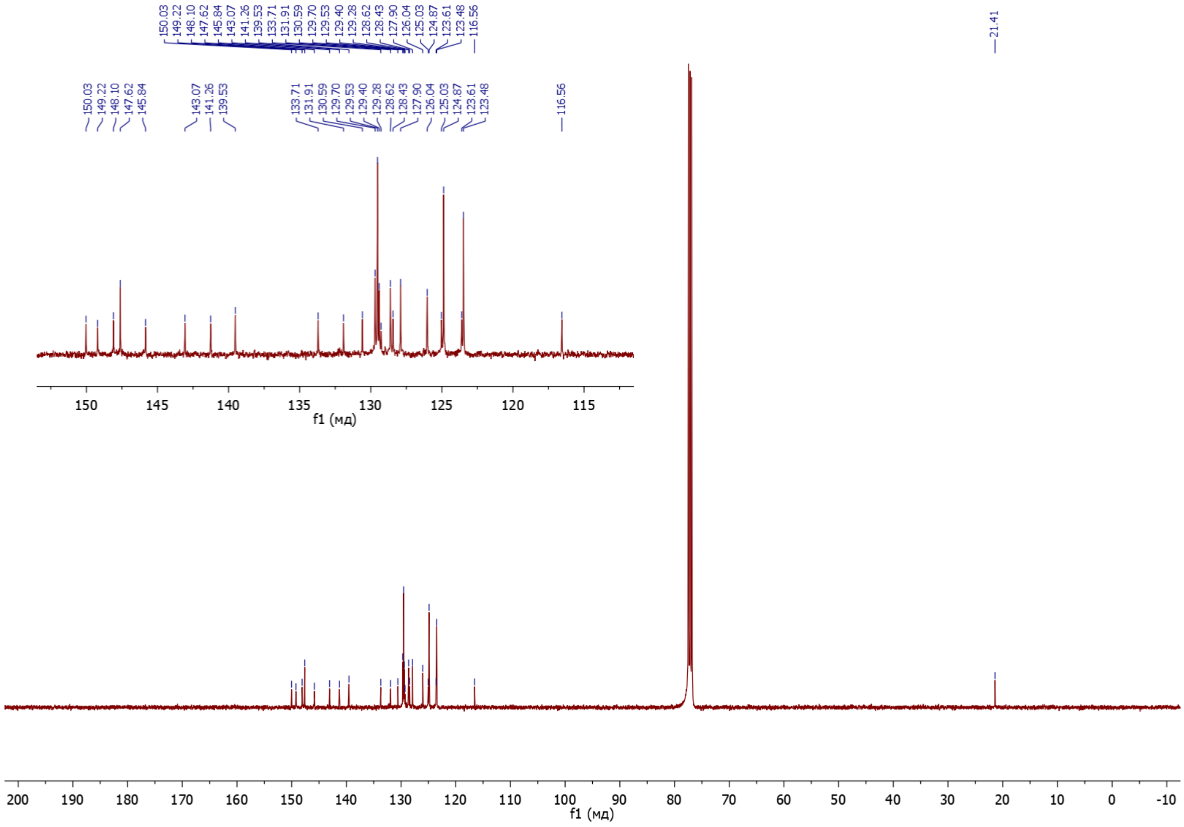

b

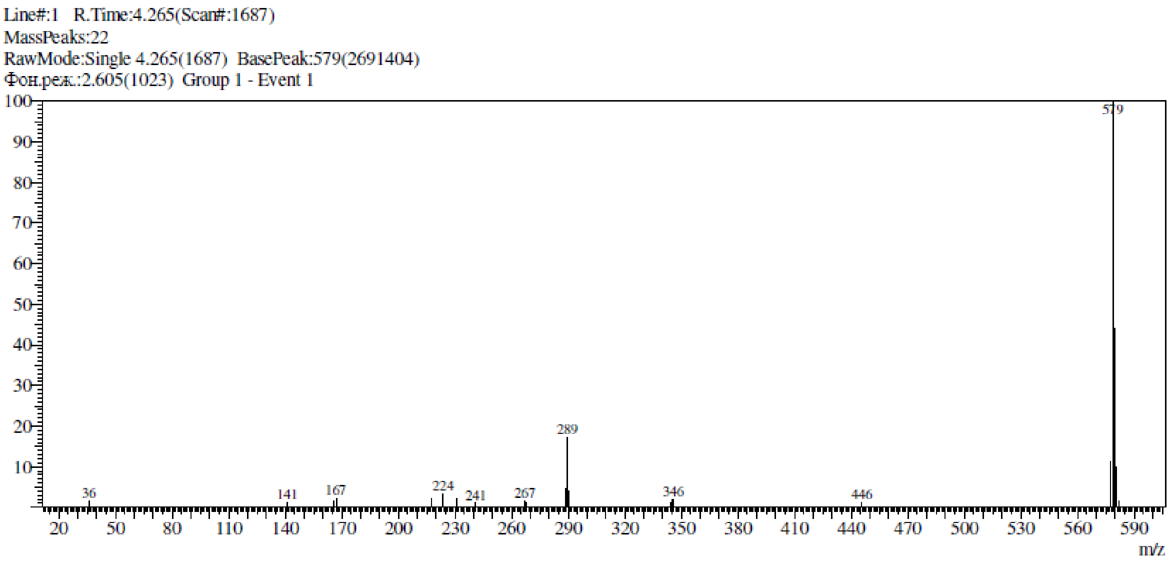

c

Figure S2. ^{1}H NMR spectrum (a) and ^{13}C NMR spectrum in $CDCl_3$ (b); mass spectrum (c) of **2b**.

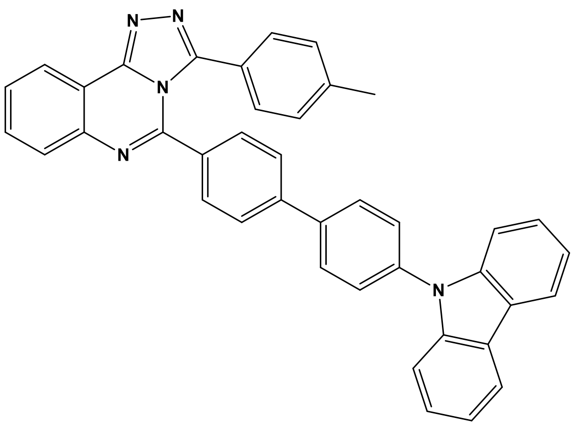

2c

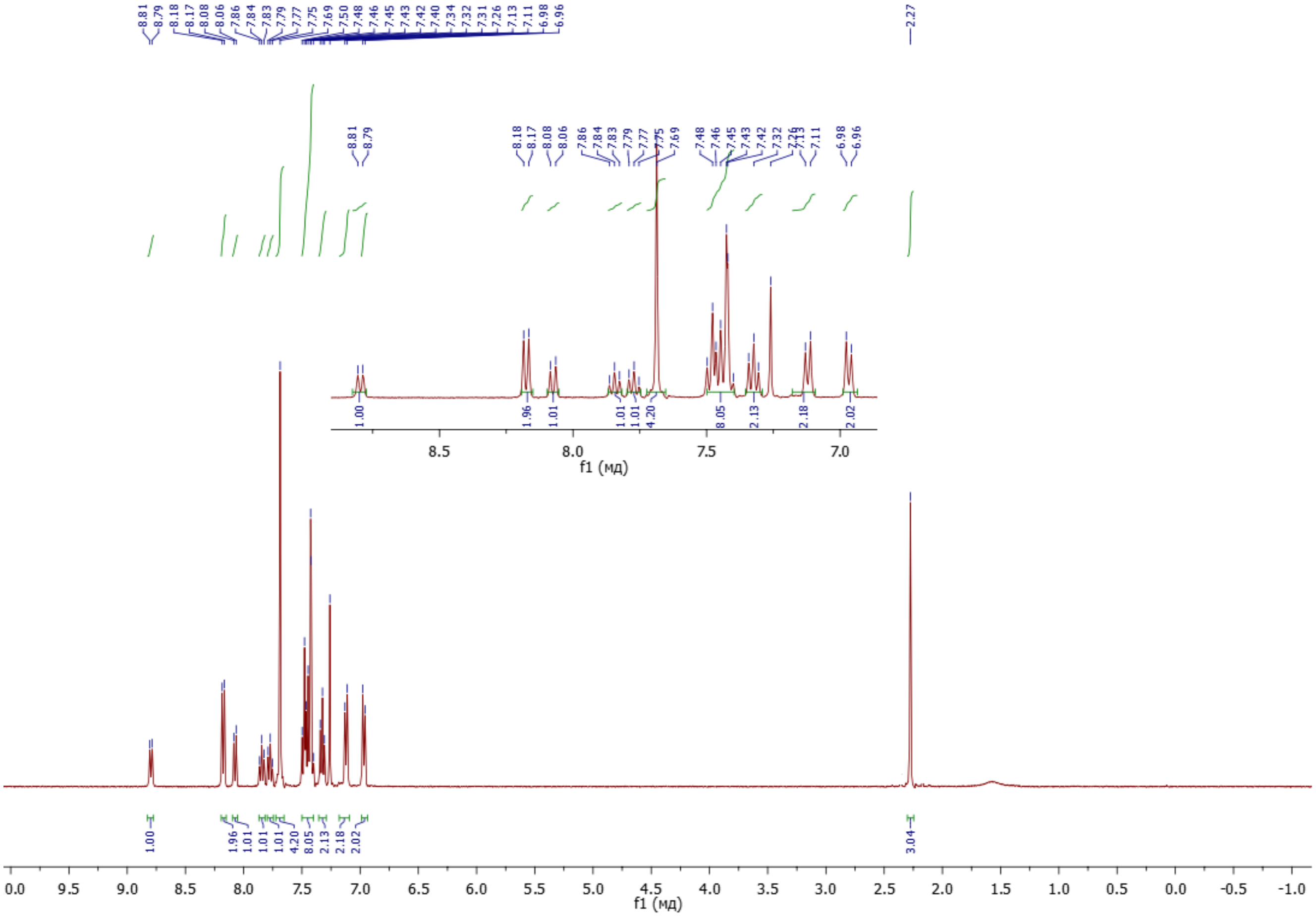

a

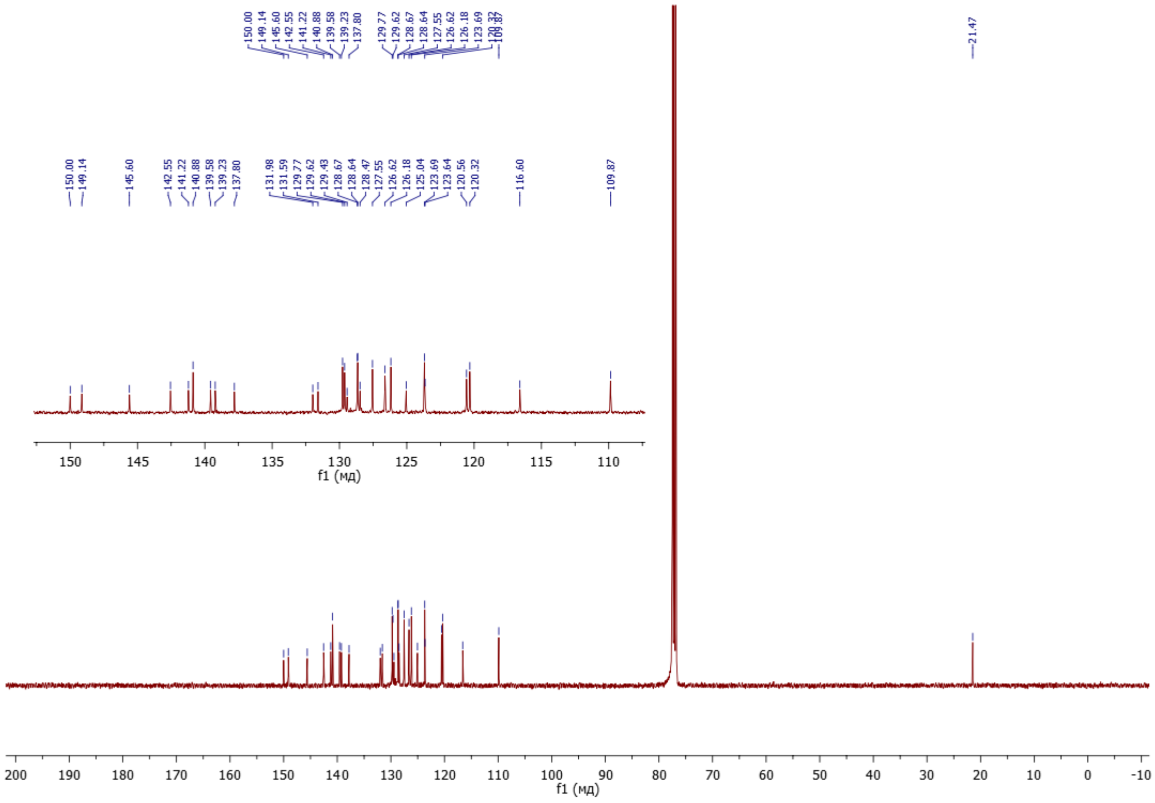

b

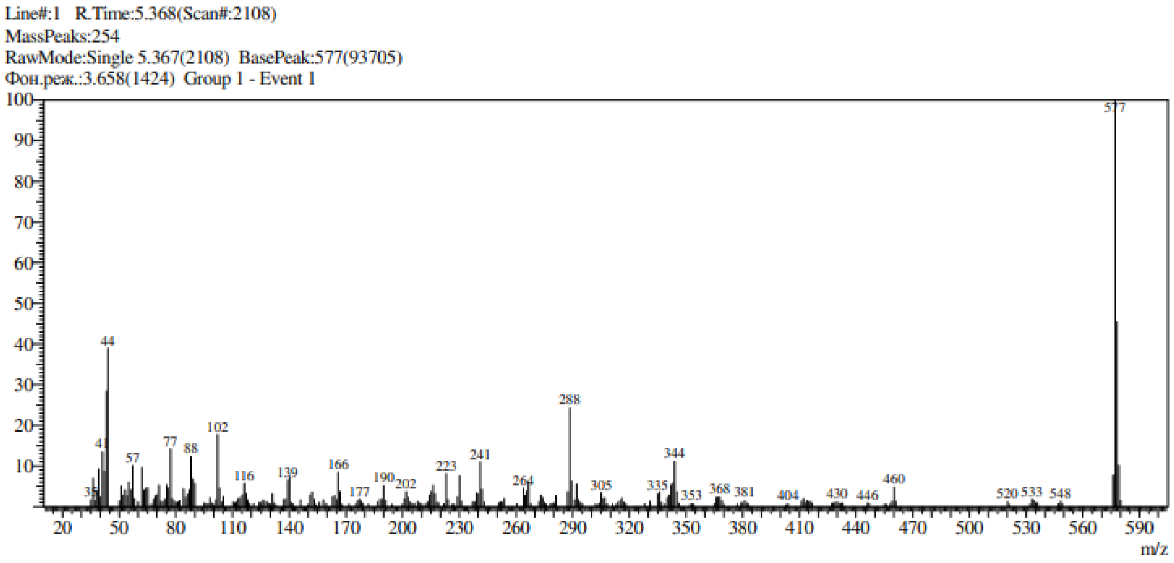

c

Figure S3. ^{1}H NMR spectrum in $CDCl_3$ (a) and ^{13}C NMR spectrum in $CDCl_3$ (b); mass spectrum (c) of **2c**.

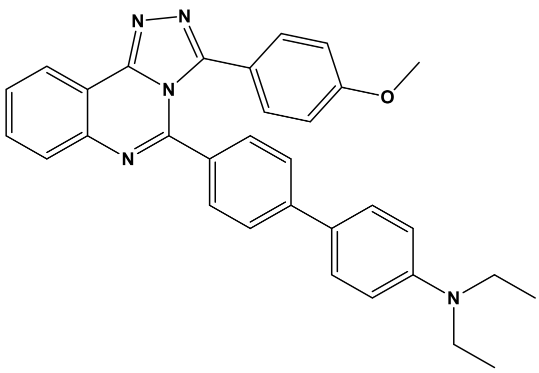

2d

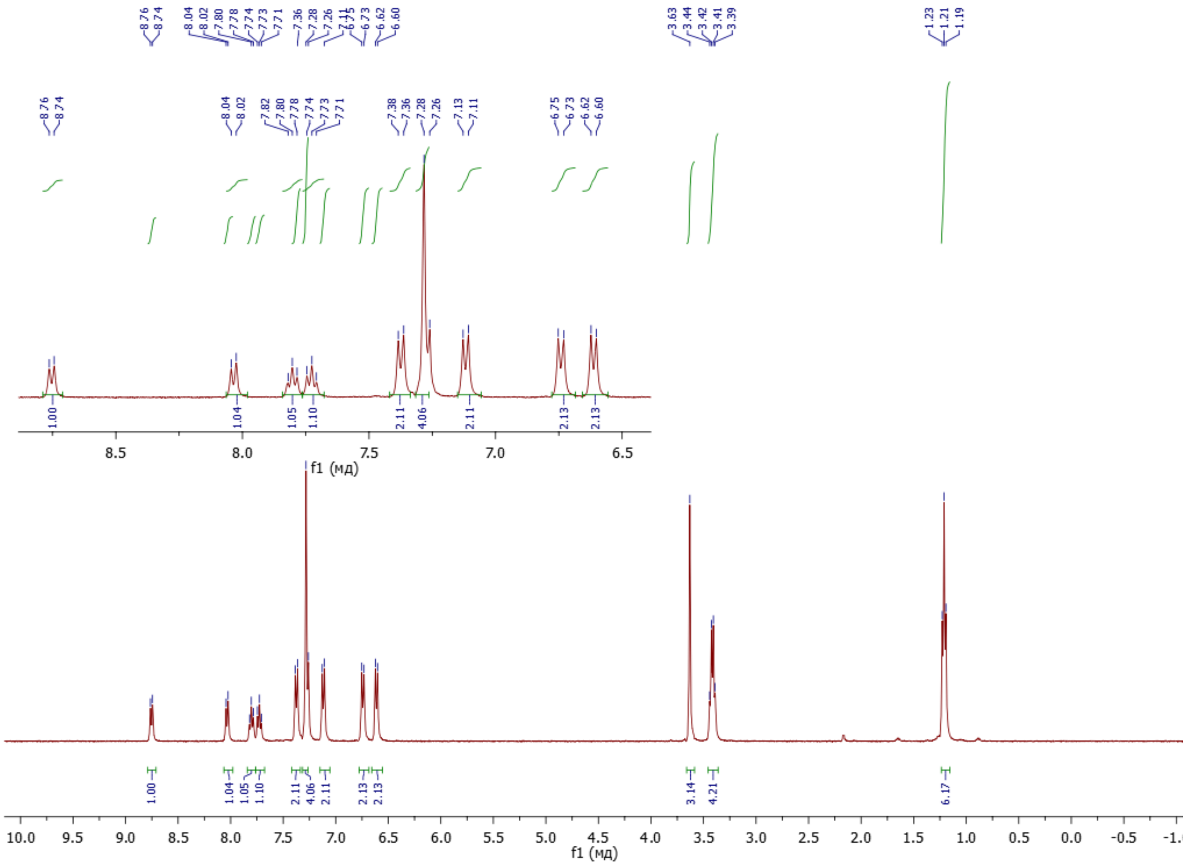

a

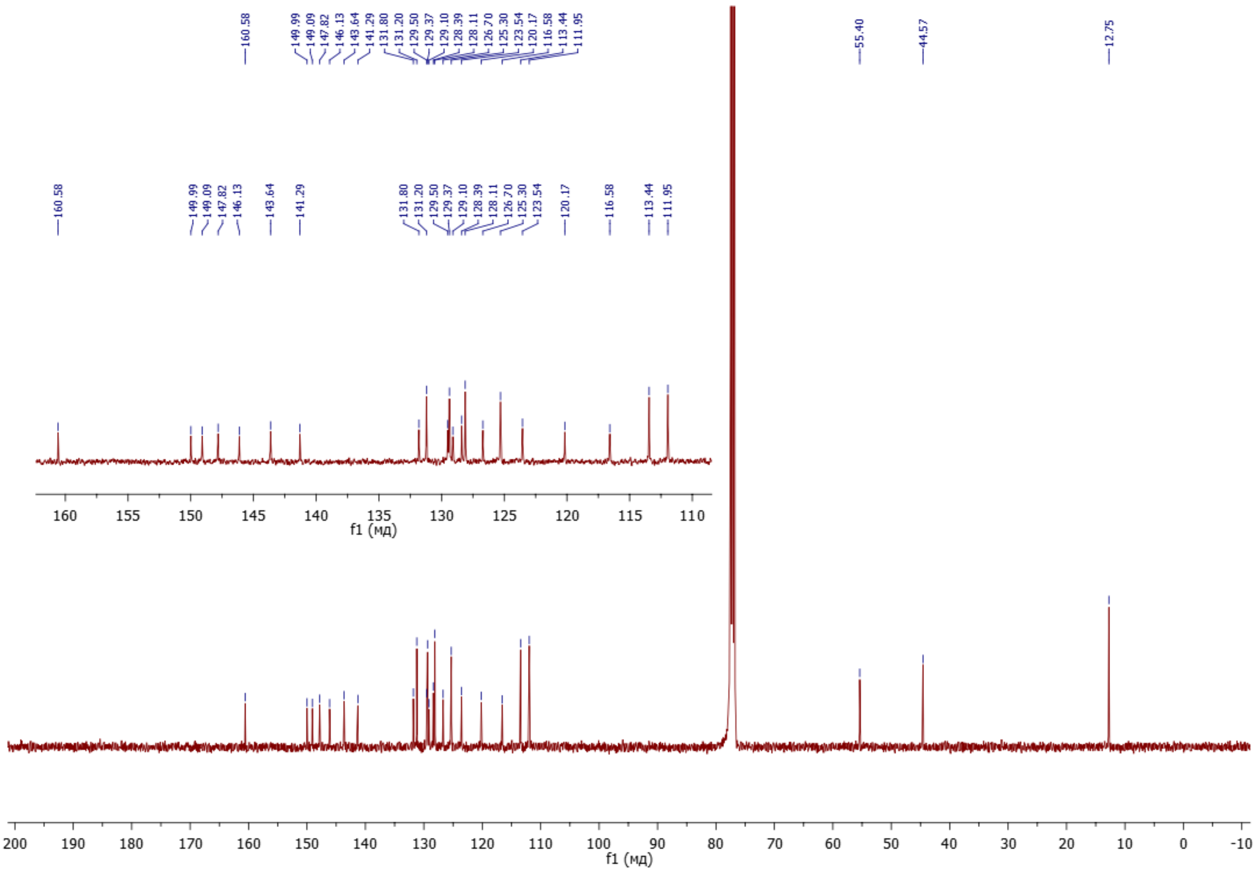

b

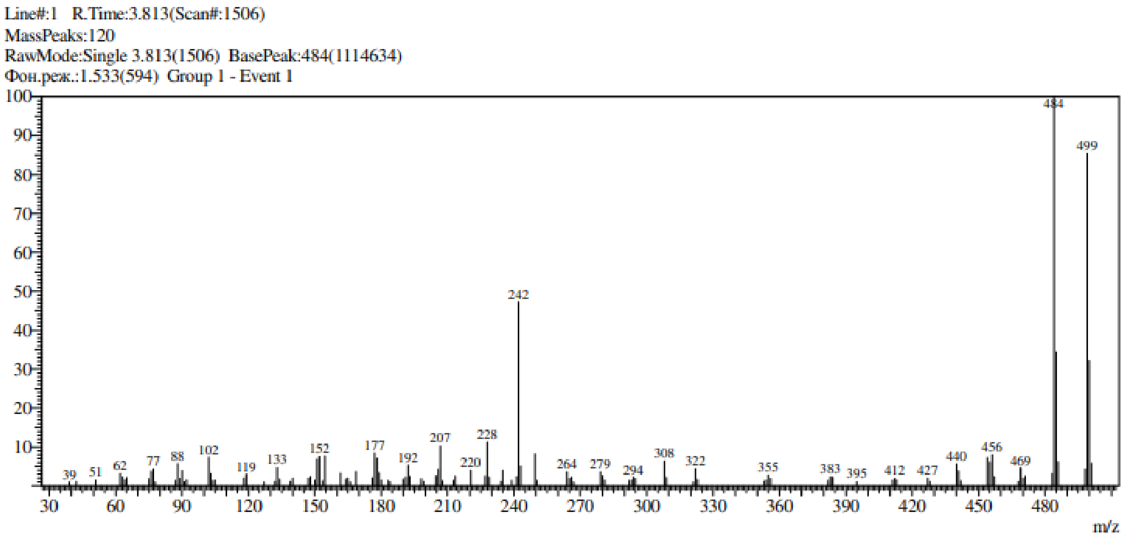

c

Figure S4. ^{1}H NMR spectrum in $CDCl_3$ (a) and ^{13}C NMR spectrum in $CDCl_3$ (b); mass spectrum (c) of **2d**.

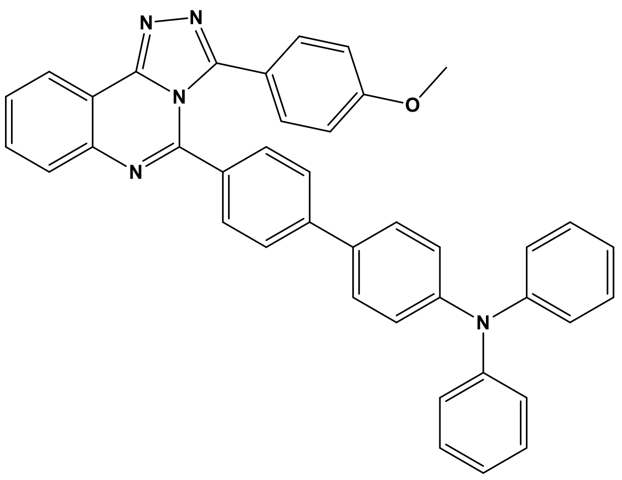

2e

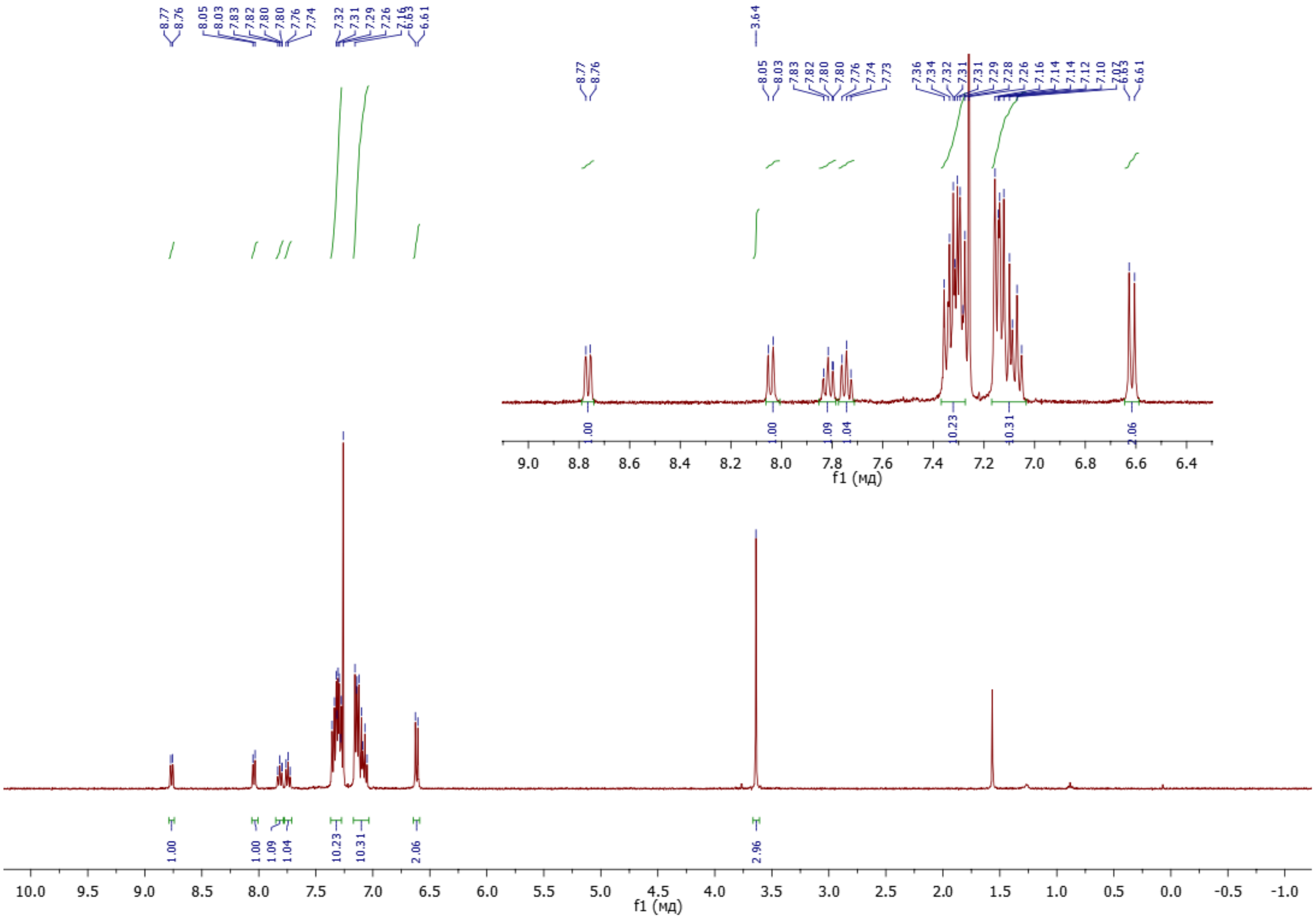

a

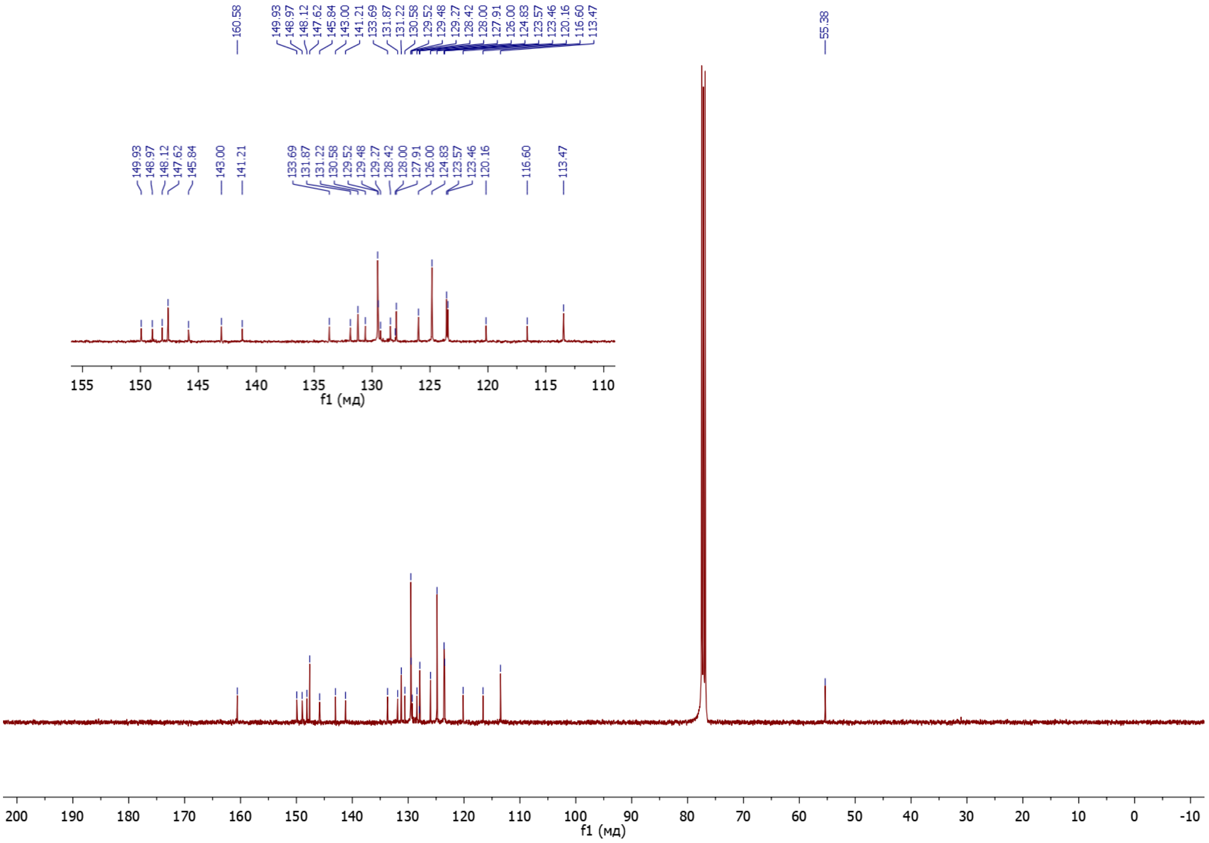

b

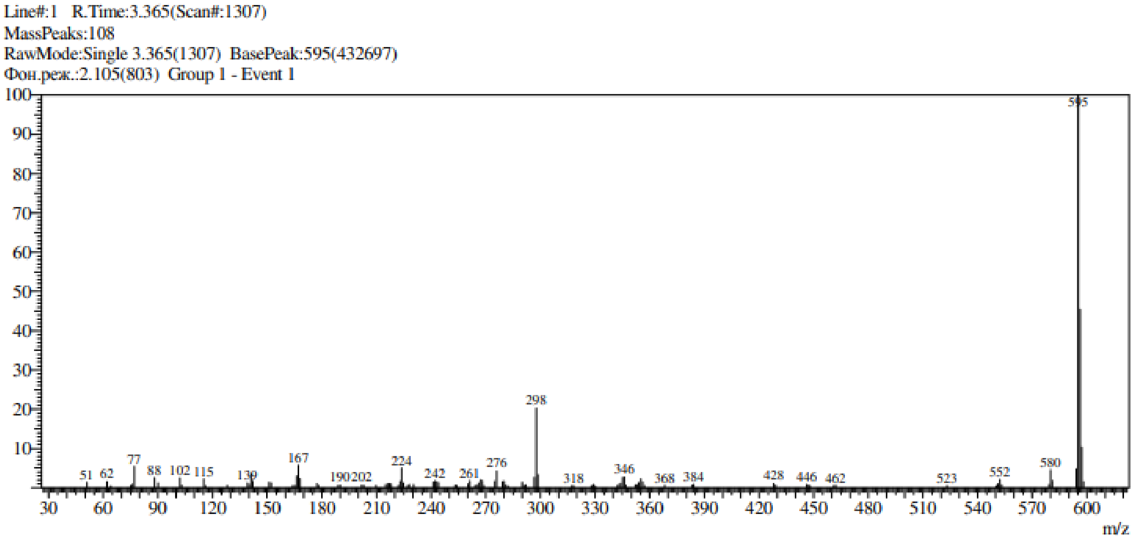

c

Figure S5. ^{1}H NMR spectrum (a) and ^{13}C NMR spectrum in $CDCl_3$ (b); mass spectrum (c) of **2e**.

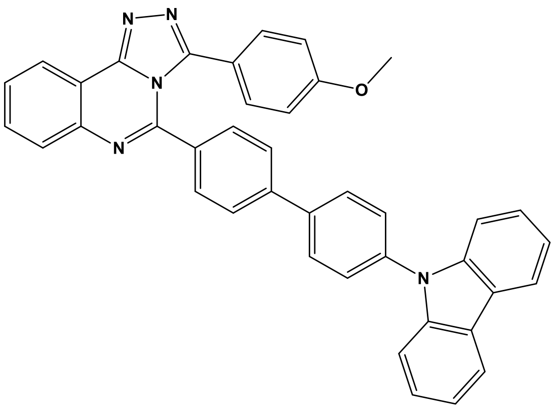

2f

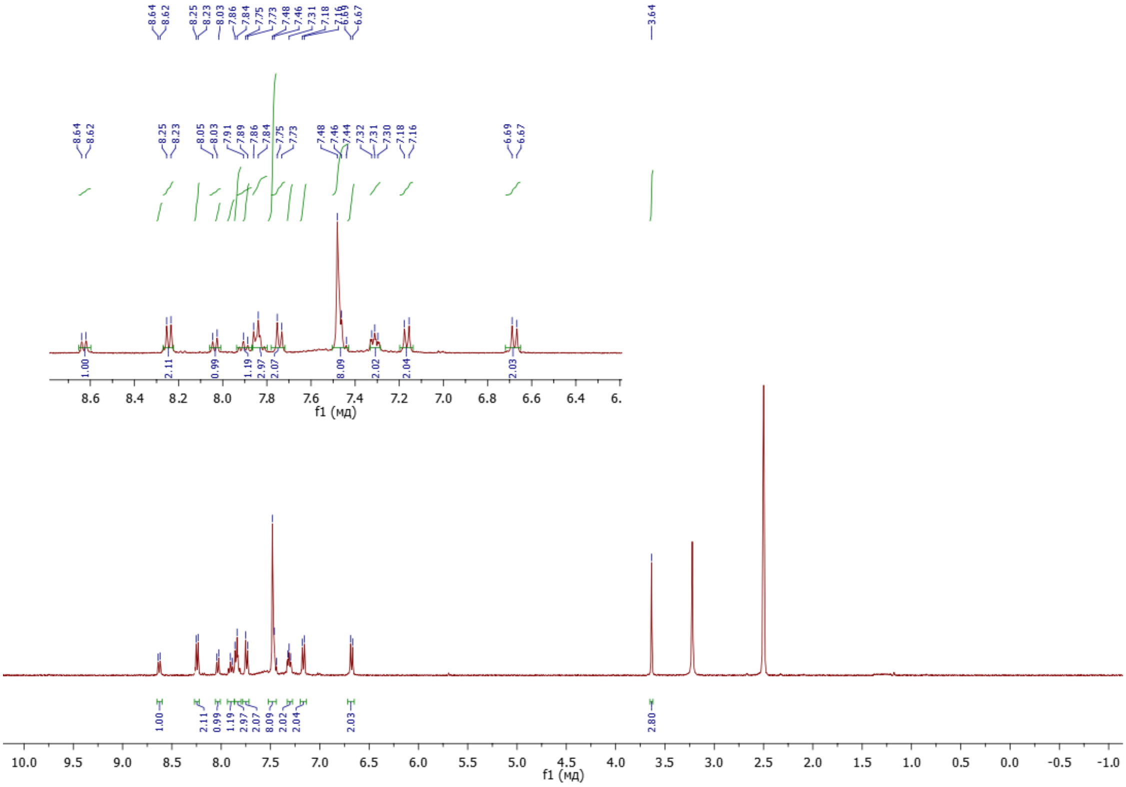

a

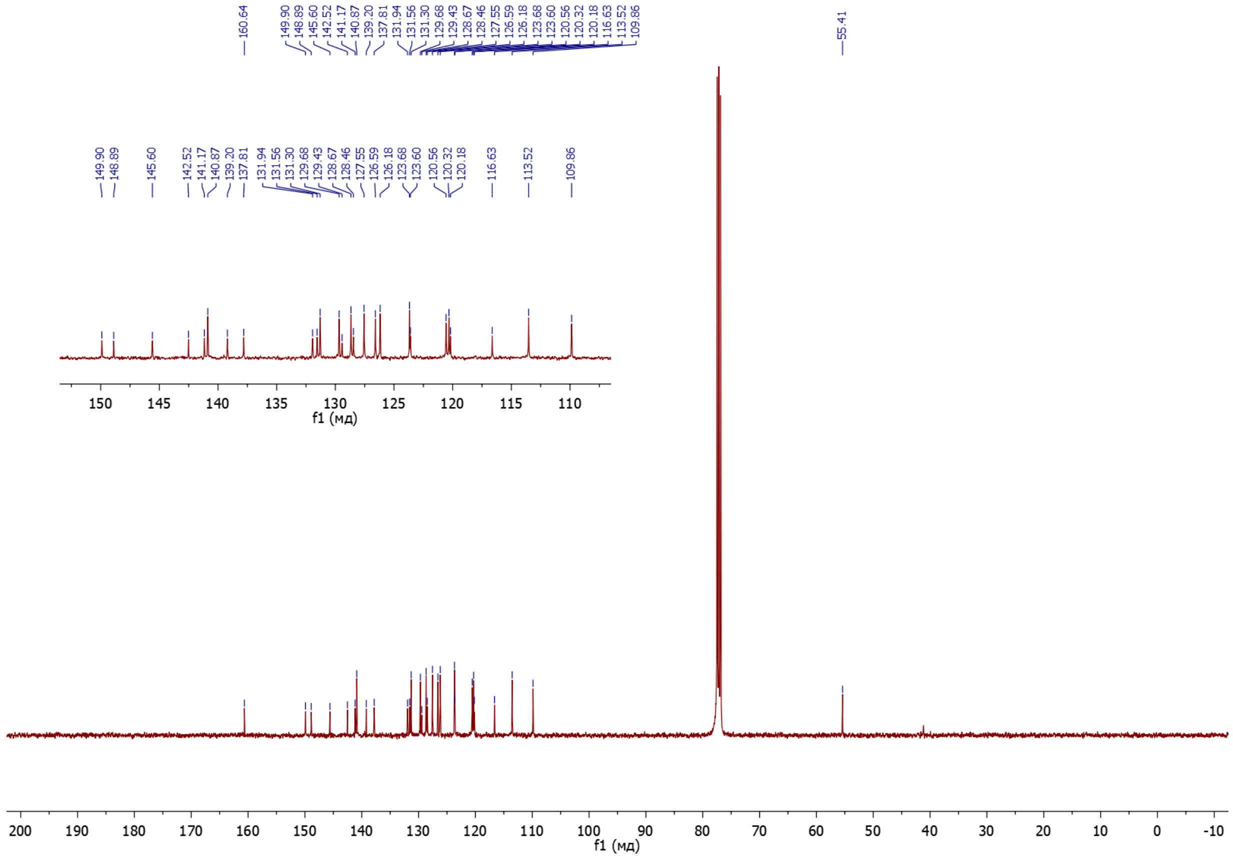

b

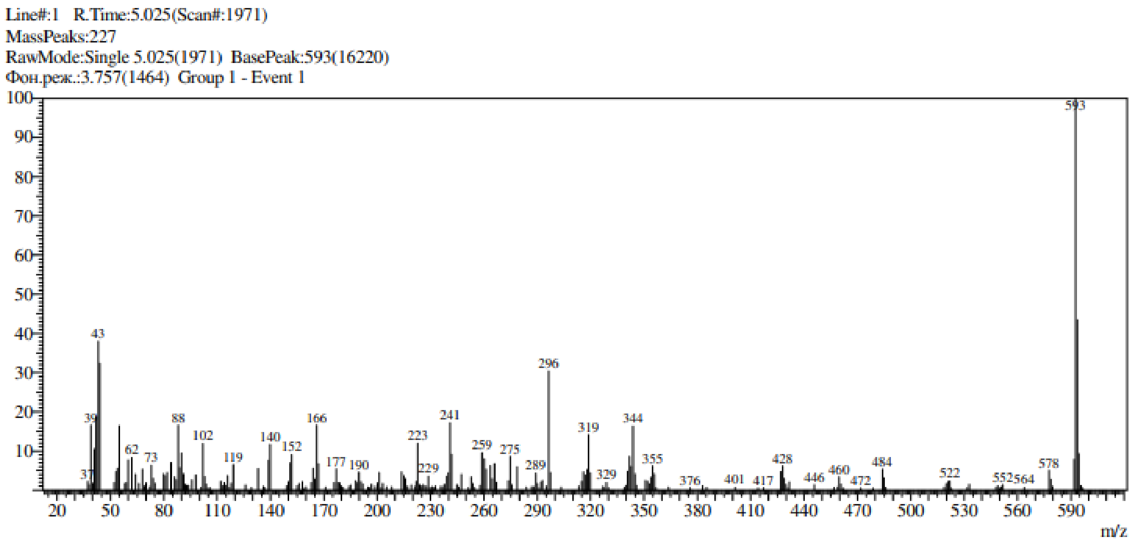

c

Figure S6. ^{1}H NMR spectrum in DMSO-d_6 (a) and ^{13}C NMR spectrum in $CDCl_3$ (b); mass spectrum (c) of **2f**.

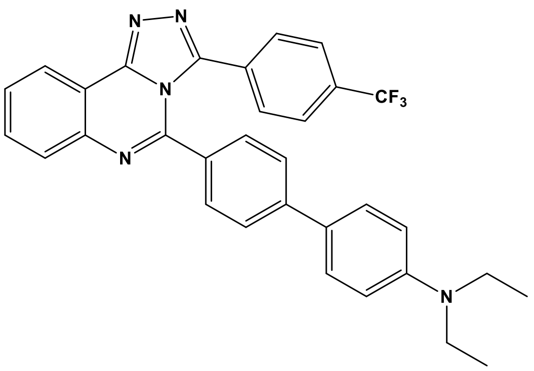

2g

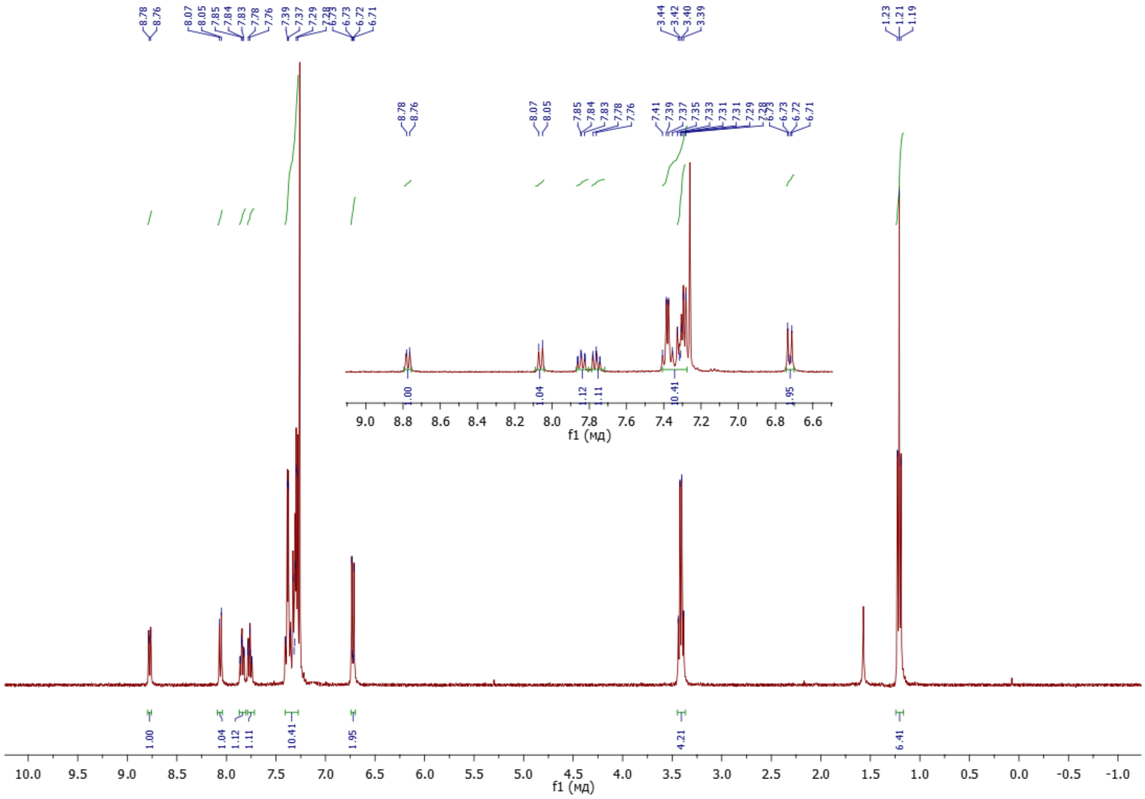

a

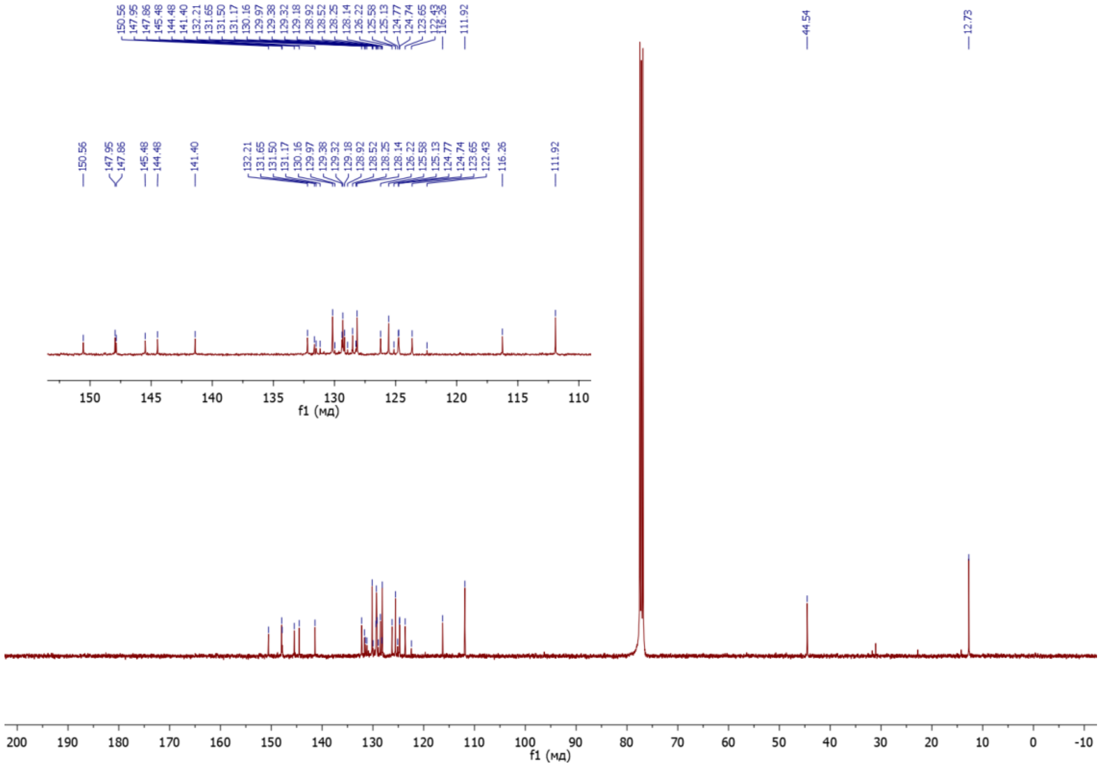
150.56
147.95
147.86
145.48
144.48
141.40
132.21
131.65
131.50
131.17
130.16
129.97
129.38
129.32
129.18
128.92
128.52
128.25
128.14
126.22
125.58
125.13
124.77
124.74
123.65
122.43
116.26
111.92
44.54
12.73
f1 (мд)

b

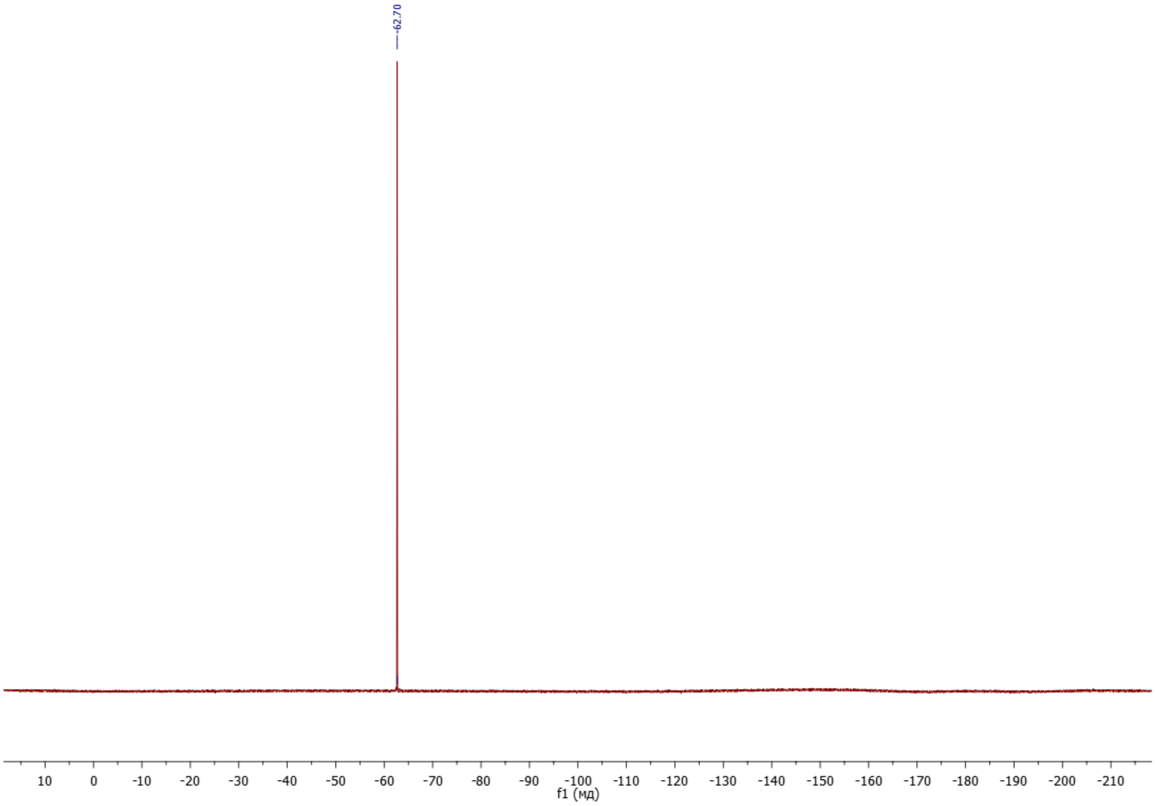
-62.70
f1 (мд)

c

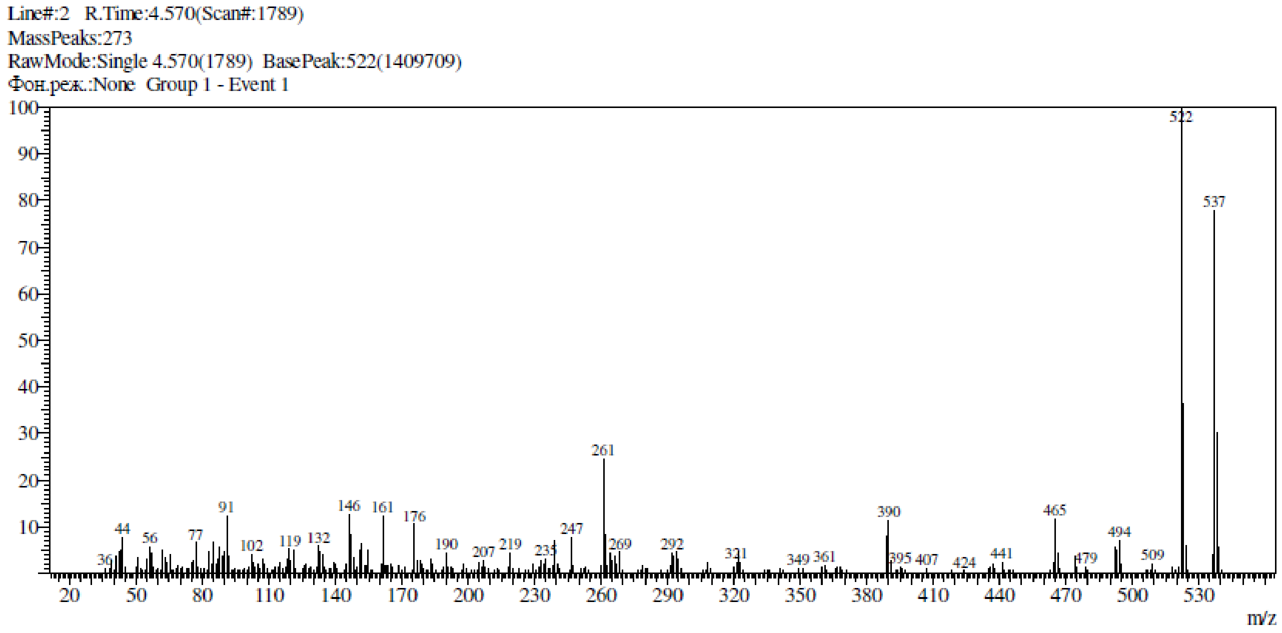

d

Figure S7. ^{1}H NMR spectrum (a), ^{13}C NMR spectrum (b), ^{19}F NMR spectrum in $CDCl_3$ (c) and mass spectrum (d) of **2g**.

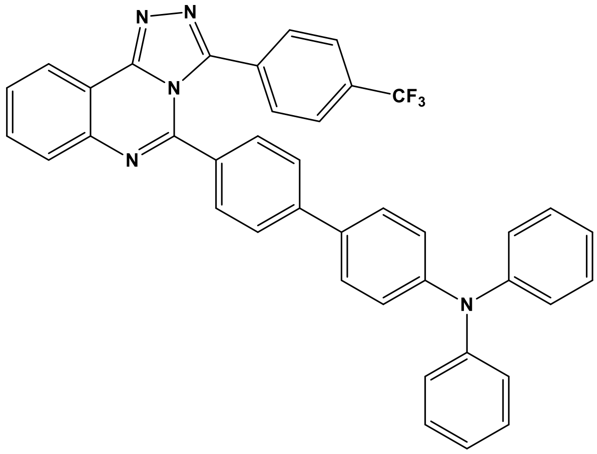

2h

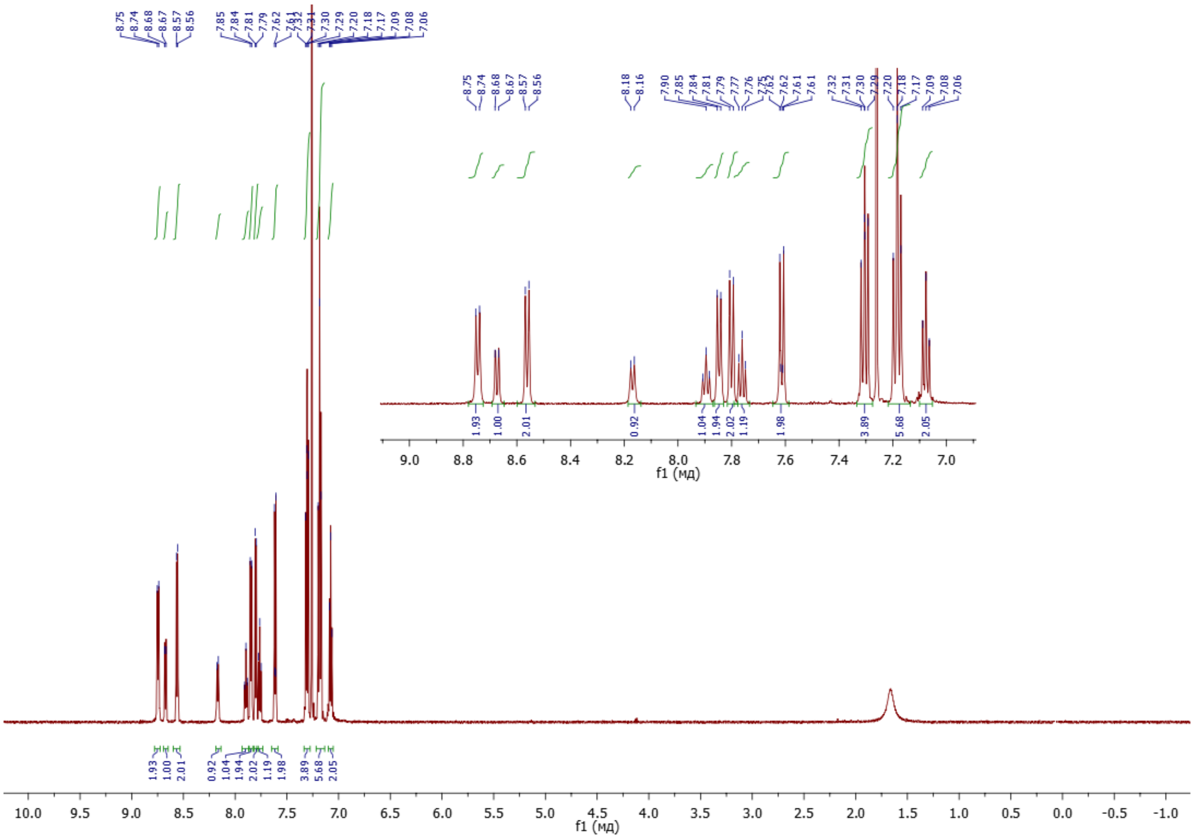
8.75 8.74 8.68 8.67 8.57 8.56 7.85 7.84 7.81 7.79 7.62 7.61 7.32 7.31 7.30 7.29 7.20 7.18 7.17 7.09 7.08 7.06
1.93 1.00 2.01 0.92 1.04 1.94 2.02 1.19 1.98 3.89 5.68 2.05
f1 (мд)
8.75 8.74 8.68 8.67 8.57 8.56 8.18 8.16 7.90 7.85 7.84 7.81 7.79 7.77 7.76 7.75 7.62 7.61 7.61 7.32 7.31 7.30 7.29 7.20 7.18 7.17 7.09 7.08 7.06
1.93 1.00 2.01 0.92 1.04 1.94 2.02 1.19 1.98 3.89 5.68 2.05
f1 (мд)

a

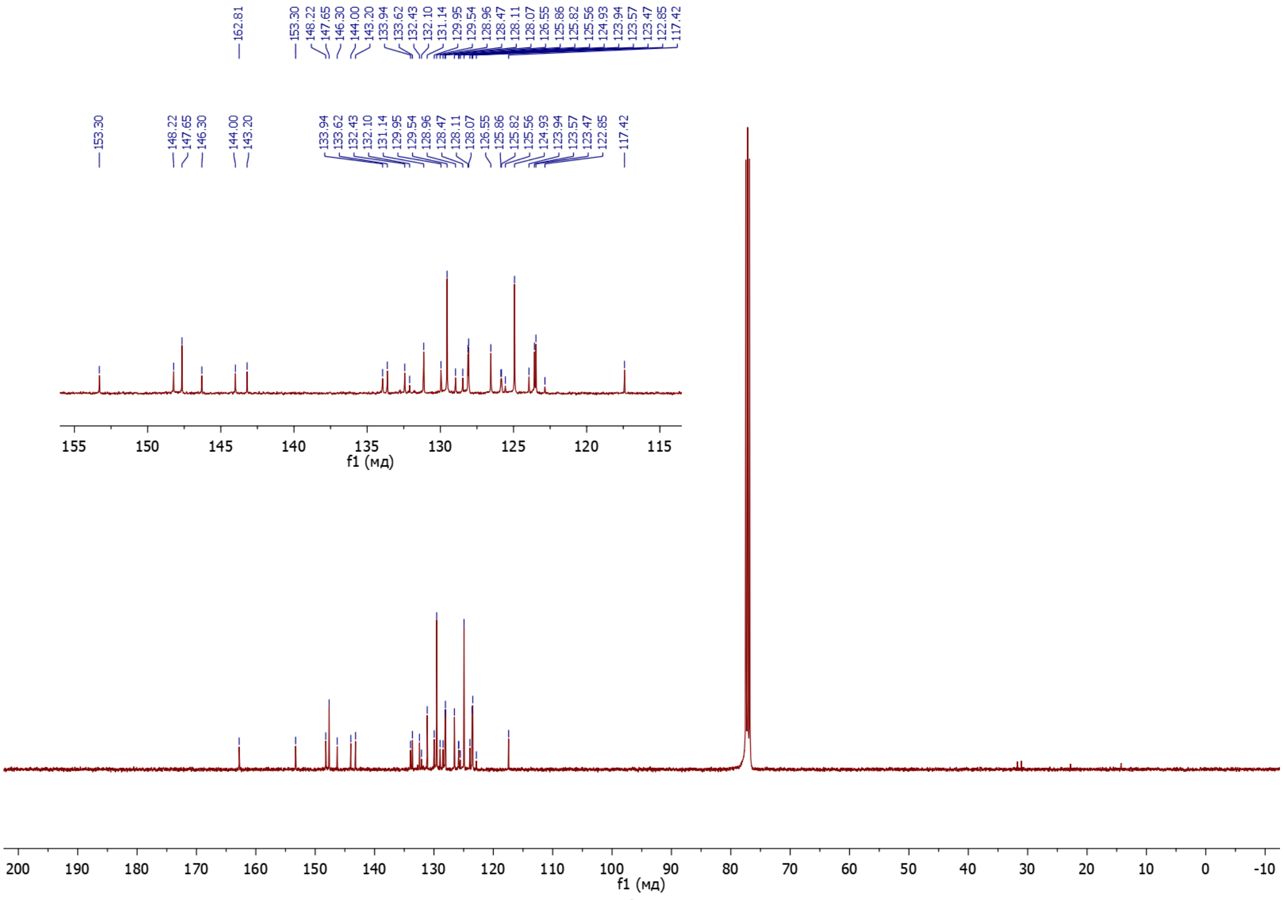
162.81 153.30 148.22 147.65 146.30 144.00 143.20 133.94 133.62 132.43 132.10 131.14 129.95 129.54 128.96 128.47 128.11 128.07 126.55 125.86 125.82 125.56 124.93 123.94 123.57 123.47 122.85 117.42
f1 (мд)
153.30 148.22 147.65 146.30 144.00 143.20 133.94 133.62 132.43 132.10 131.14 129.95 129.54 128.96 128.47 128.11 128.07 126.55 125.86 125.82 125.56 124.93 123.94 123.57 123.47 122.85 117.42
f1 (мд)

b

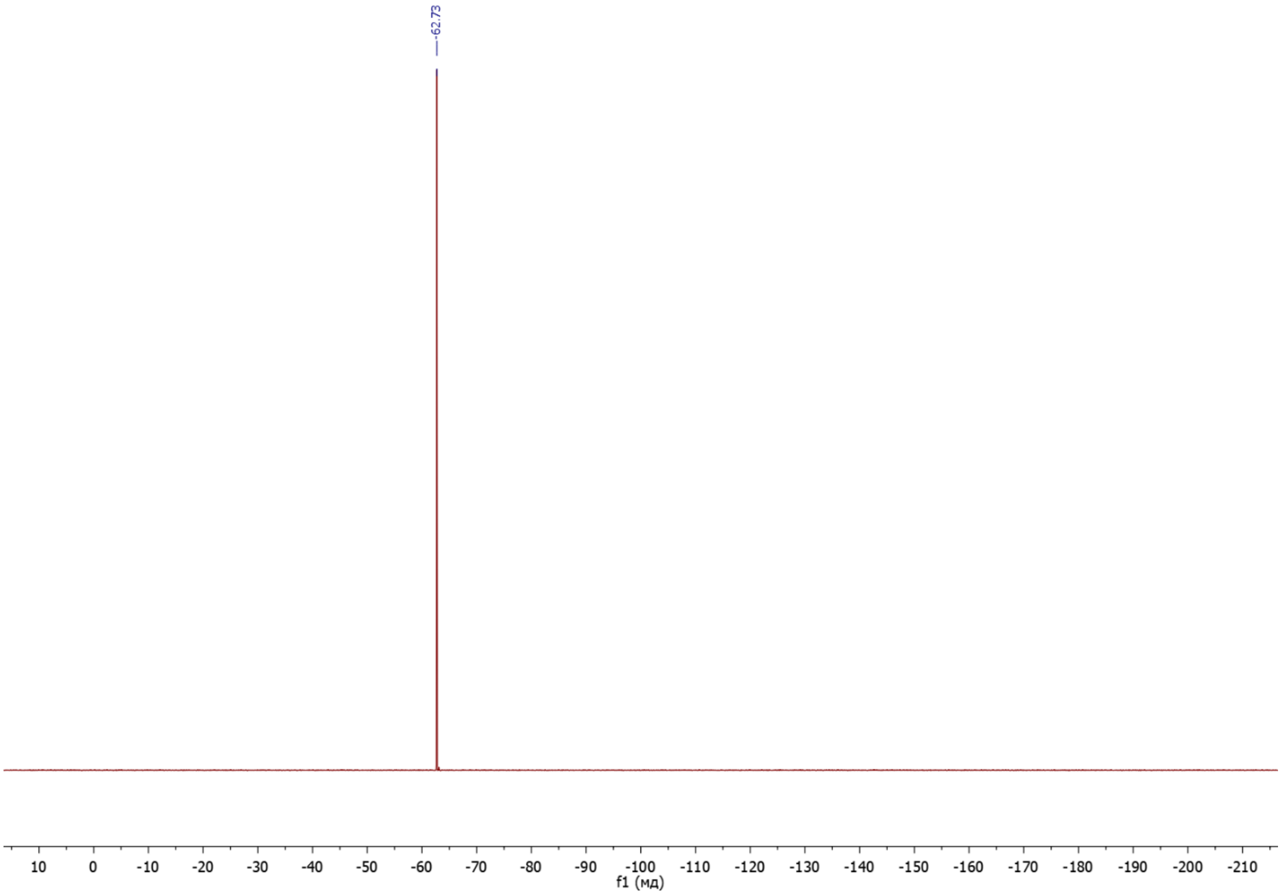

c

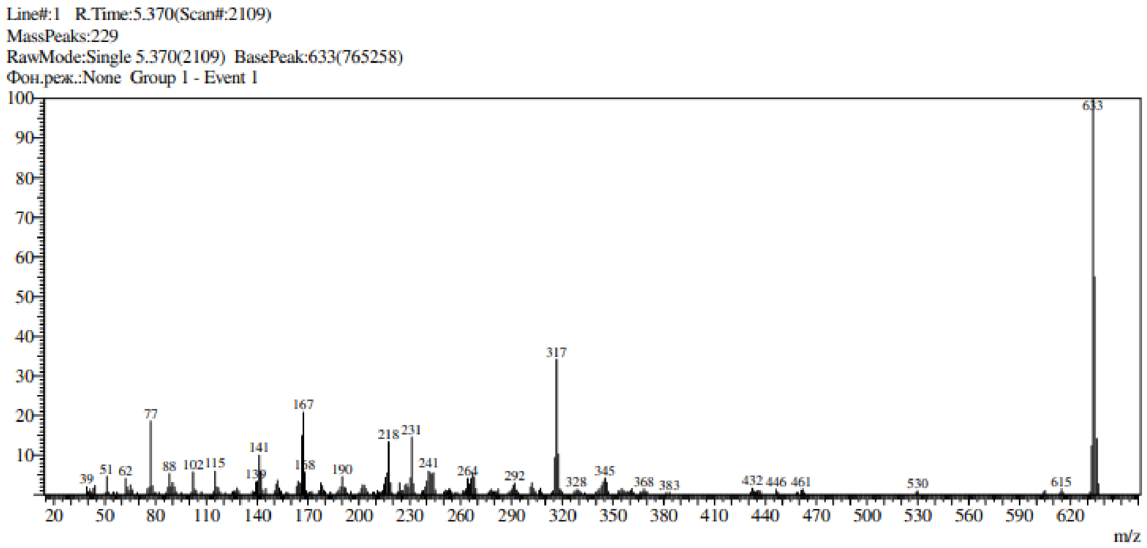

d

Figure S8. ^{1}H NMR spectrum (a), ^{13}C NMR spectrum (b), ^{19}F NMR spectrum in $CDCl_3$ (c) and mass spectrum (d) of **2h**.

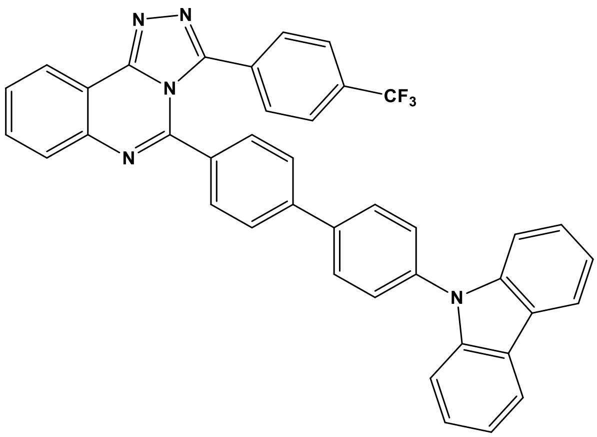

2i

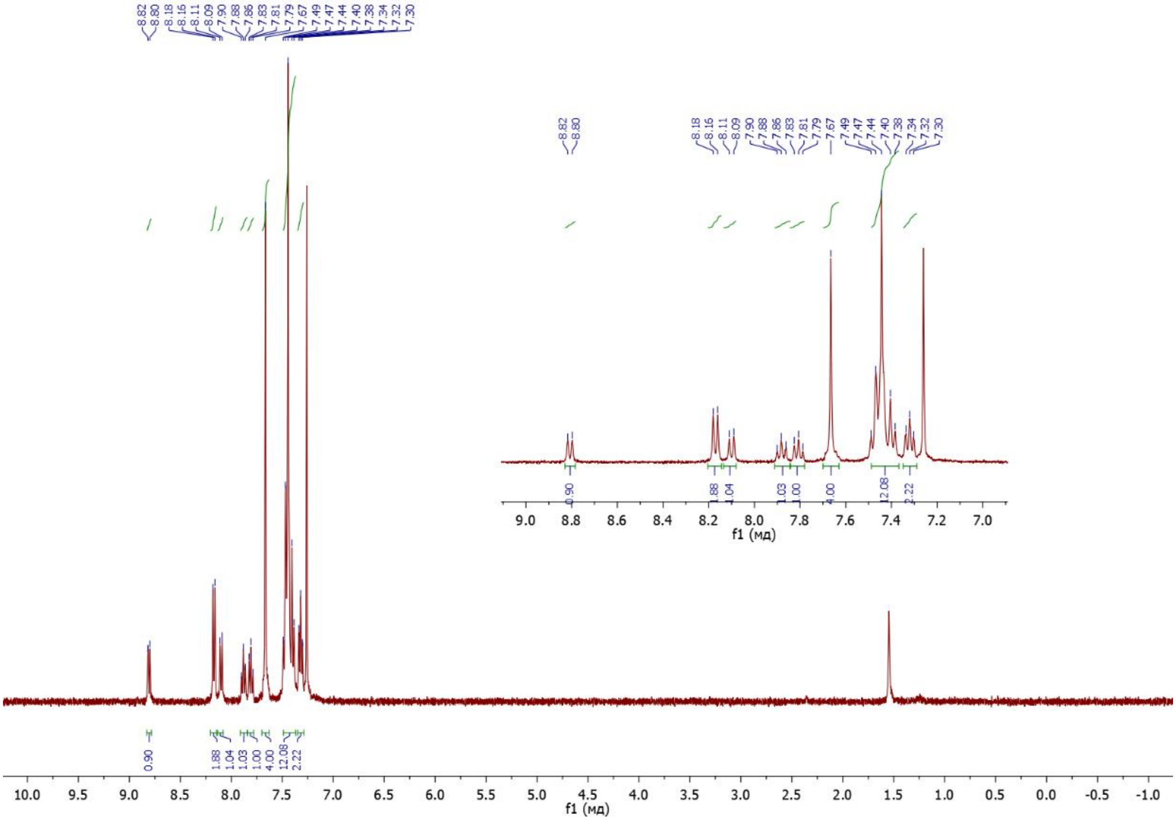
8.82
8.80
8.18
8.16
8.11
8.09
7.90
7.88
7.86
7.83
7.81
7.79
7.67
7.49
7.47
7.44
7.40
7.38
7.34
7.32
7.30
0.90
1.88
1.04
1.03
1.00
4.00
12.08
2.22
f1 (мд)

a

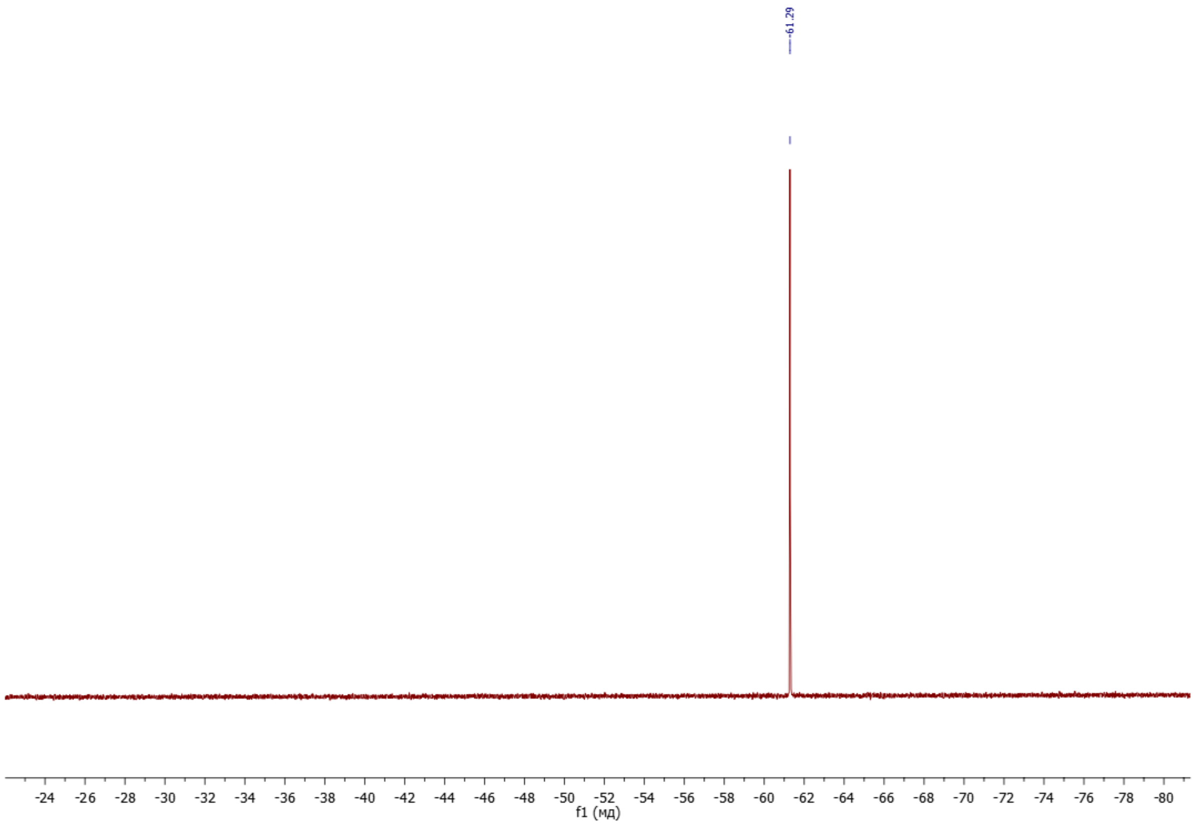

b

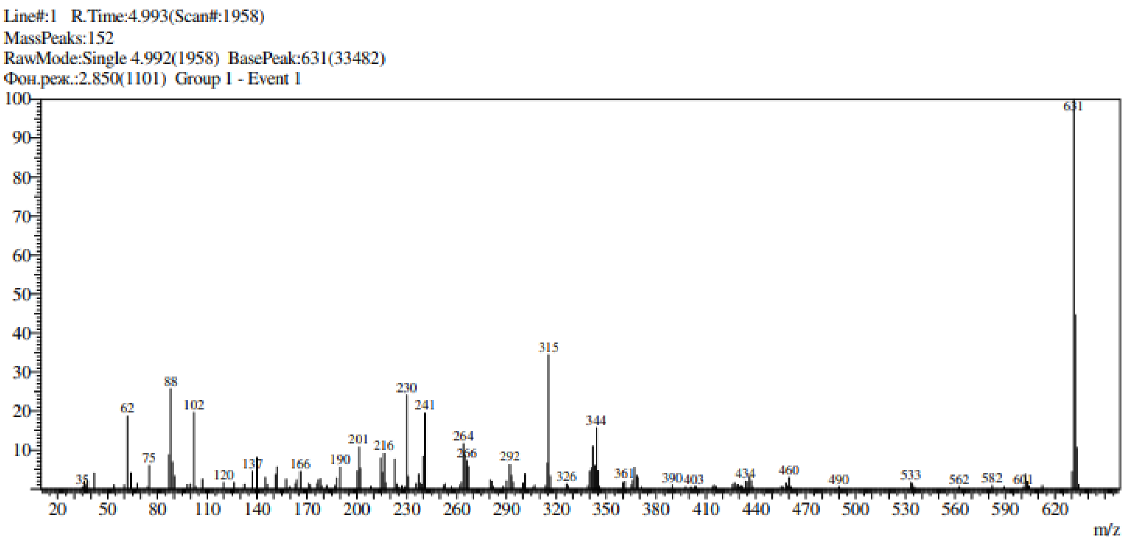

c

Figure S9. ^{1}H NMR spectrum in $CDCl_3$ (a), ^{19}F NMR spectrum in $CDCl_3$ (b); mass spectrum (c) of **2i**.

2. Crystallographic data of compounds 2a and 2e

Table S1. Selected bond lengths of compound **2a**.

**Bond**	**Bond length (Å)**	**Bond**	**Bond length (Å)**
N(13) – C(4)	1.380(4)	C(10) – C(5)	1.402(5)
N(13) – C(12)	1.418(4)	C(10) – C(9)	1.394(5)
N(13) – C(1)	1.393(4)	C(24) – C(23)	1.381(5)
N(11) – C(10)	1.405(4)	C(1) – C(14)	1.467(5)
N(11) – C(12)	1.291(4)	C(5) – C(6)	1.399(5)
N(2) – N(3)	1.398(5)	C(29) – C(30)	1.380(5)
N(2) – C(1)	1.309(5)	C(31) – C(30)	1.404(5)
N(3) – C(4)	1.306(4)	C(31) – C(32)	1.397(5)
N(34) – C(31)	1.386(5)	C(14) – C(15)	1.390(5)
N(34) – C(35)	1.456(5)	C(14) – C(19)	1.389(5)
N(34) – C(37)	1.461(6)	C(15) – C(16)	1.378(5)
N(27) – C(22)	1.396(5)	C(33) – C(32)	1.375(5)
N(27) – C(26)	1.380(5)	C(6) – C(7)	1.366(5)
C(22) – C(12)	1.468(5)	C(9) – C(8)	1.372(5)
C(22) – C(23)	1.395(5)	C(16) – C(17)	1.388(6)
C(25) – C(28)	1.481(5)	C(7) – C(8)	1.396(6)
C(25) – C(24)	1.388(5)	C(19) – C(18)	1.382(6)
C(25) – C(26)	1.403(5)	C(17) – C(18)	1.375(6)
C(4) – C(5)	1.433(5)	C(17) – C(20)	1.511(6)
C(28) – C(29)	1.382(5)	C(35) – C(36)	1.493(7)
C(28) – C(33)	1.391(5)	C(37) – C(38)	1.485(7)

Table S2. Selected bond angles of compound **2a**.

**Angle**	**(°)**	**Angle**	**(°)**
C(4) – N(13) – C(12)	120.9(3)	N(6) – C(1) – N(13)	109.1(3)
C(4) – N(13) – C(1)	104.3(3)	N(2) – C(1) – C(14)	122.9(3)
C(1) – N(13) – C(12)	134.6(3)	C(10) – C(5) – C(4)	116.2(3)
C(12) – N(11) – C(10)	120.3(3)	C(6) – C(5) – C(4)	123.8(3)
C(1) – N(2) – N(3)	108.9(3)	C(6) – C(5) – C(10)	120.0(3)
C(4) – N(3) – N(2)	106.3(3)	C(27) – C(26) – C(25)	121.4(3)

C(31) – N(34) – C(35)	122.1(3)	C(24) – C(23) – C(22)	120.4(3)
C(31) – N(34) – C(37)	120.8(3)	C(30) – C(29) – C(28)	122.1(3)
C(35) – N(34) – C(37)	115.7(3)	N(34) – C(31) – C(30)	122.6(3)
C(26) – C(27) – C(22)	120.4(3)	N(34) – C(31) – C(32)	121.0(3)
C(27) – C(22) – C(12)	121.4(3)	C(32) – C(31) – C(30)	116.4(3)
C(23) – C(22) – C(27)	118.5(3)	C(15) – C(14) – C(1)	119.9(3)
C(23) – C(22) – C(12)	120.1(3)	C(19) – C(14) – C(1)	121.2(3)
C(24) – C(25) – C(28)	122.6(3)	C(19) – C(14) – C(15)	118.9(4)
C(24) – C(25) – C(26)	117.4(3)	C(29) – C(30) – C(31)	121.1(3)
C(26) – C(25) – C(28)	120.0(3)	C(16) – C(15) – C(14)	119.8(4)
N(13) – C(4) – C(5)	118.8(3)	C(32) – C(33) – C(28)	121.6(3)
N(3) – C(4) – N(13)	111.3(3)	C(7) – C(6) – C(5)	119.8(4)
N(3) – C(4) – C(5)	129.9(3)	C(8) – C(9) – C(10)	120.1(4)
C(29) – C(28) – C(25)	121.8(3)	C(33) – C(32) – C(31)	121.8(3)
C(29) – C(28) – C(33)	117.0(3)	C(15) – C(16) – C(17)	121.6(4)
C(33) – C(28) – C(25)	121.3(3)	C(6) – C(7) – C(8)	120.4(4)
C(5) – C(10) – N(11)	122.2(3)	C(18) – C(19) – C(14)	120.3(4)
C(9) – C(10) – N(11)	118.3(3)	C(9) – C(8) – C(7)	120.5(4)
C(9) – C(10) – C(5)	119.2(3)	C(16) – C(17) – C(20)	121.1(4)
N(13) – C(12) – C(22)	120.2(3)	C(18) – C(17) – C(16)	118.0(4)
N(11) – C(12) – N(13)	120.3(3)	C(18) – C(17) – C(20)	120.9(4)
N(11) – C(12) – C(22)	119.4(3)	C(17) – C(18) – C(19)	121.3(4)
C(23) – C(24) – C(25)	121.8(3)	N(34) – C(35) – C(36)	114.7(4)
N(13) – C(1) – C(14)	127.7(3)	N(34) – C(37) – C(38)	113.6(5)

Table S3. Selected bond lengths of compound **2e**.

**Bond**	**Bond length (Å)**	**Bond**	**Bond length (Å)**
N(13) – C(1)	1.401(6)	C(28) – C(33)	1.398(7)
N(13) – C(12)	1.412(6)	C(28) – C(29)	1.385(7)
N(13) – C(4)	1.388(6)	C(15) – C(16)	1.379(7)
O(20) – C(17)	1.374(6)	C(17) – C(18)	1.367(8)
O(20) – C(21)	1.412(9)	C(17) – C(16)	1.376(8)
N(2) – N(3)	1.409(6)	C(39) – C(40)	1.387(8)

N(2) – C(1)	1.306(6)	C(39) – C(44)	1.380(7)
N(11) – C(10)	1.392(7)	C(33) – C(32)	1.373(7)
N(11) – C(12)	1.292(6)	C(5) – C(6)	1.403(7)
N(34) – C(31)	1.409(6)	C(40) – C(41)	1.382(8)
N(34) – C(39)	1.423(6)	C(19) – C(18)	1.378(7)
N(34) – C(35)	1.442(7)	C(24) – C(23)	1.376(7)
N(3) – C(4)	1.300(7)	C(7) – C(6)	1.374(8
C(14) – C(1)	1.477(7)	C(7) – C(8)	1.390(9)
C(14) – C(15)	1.365(8)	C(44) – C(43)	1.384(8)
C(14) – C(19)	1.380(7)	C(30) – C(29)	1.385(7)
C(10) – C(5)	1.400(7)	C(43) – C(42)	1.371(9)
C(10) – C(9)	1.400(8)	C(42) – C(41)	1.382(8)
C(12) – C(22)	1.475(7)	C(8) – C(9)	1.378(8)
C(4) – C(5)	1.436(7)	C(35) – C(36)	1.380(9)
C(26) – C(25)	1.394(7)	C(35) – C(38)	1.375(9)
C(26) – C(27)	1.383(6)	C(36) – C(37)	1.386(12)
C(25) – C(28)	1.472(7)	C(21) – H(21A)	0.9600
C(25) – C(24)	1.387(7)	C(21) – H(21B)	0.9600
C(22) – C(27)	1.388(7)	C(21) – H(21C)	0.9600
C(22) – C(23)	1.380(8)	C(37) – C(2)	1.351(16)
C(31) – C(32)	1.400(8)	C(38) – C(3)	1.402(12)
C(31) – C(30)	1.389(7)	C(2) – C(3)	1.372(17)

Table S4. Selected bond angles of compound **2e**.

**Angle**	**(°)**	**Angle**	**(°)**
C(1) – N(13) – C(12)	135.0(4)	O(20) – C(17) – C(16)	115.5(5)
C(4) – N(13) – C(1)	104.3(4)	C(18) – C(17) – O(20)	124.6(5)
C(4) – N(13) – C(12)	120.6(4)	C(18) – C(17) – C(16)	119.9(5)
C(17) – O(20) – C(21)	117.6(5)	C(40) – C(39) – N(34)	120.6(5)
C(1) – N(2) – N(3)	109.3(4)	C(44) – C(39) – N(34)	120.2(5)
C(12) – N(11) – C(10)	120.2(4	C(44) – C(39) – C(40)	119.2(5)
C(31) – N(34) – C(39)	121.7(4)	C(32) – C(33) – C(28)	121.9(5)
C(31) – N(34) – C(35)	117.2(4)	C(10) – C(5) – C(4)	116.2(4)

C(39) – N(34) – C(35)	119.6(4)	C(10) – C(5) – C(6)	120.9(5)
C(4) – N(3) – N(2)	106.1(4)	C(6) – C(5) – C(4)	122.8(5)
C(15) – C(14) – C(1)	122.3(5)	C(41) – C(40) – C(39)	119.2(5)
C(15) – C(14) – C(19)	119.6(5)	C(18) – C(19) – C(14)	120.4(5)
C(19) – C(14) – C(1)	118.1(5)	C(6) – C(7) – C(8)	120.0(5)
N(11) – C(10) – C(5)	122.8(4)	C(17) – C(18) – C(19)	119.8(5)
N(11) – C(10) – C(9)	118.1(5)	C(39) – C(44) – C(43)	121.0(5)
C(9) – C(10) – C(5)	119.1(5)	C(33) – C(32) – C(31)	120.3(5)
N(13) – C(1) – C(14)	128.0(4)	C(29) – C(30) – C(31)	120.5(5)
N(2) – C(1) – N(13)	108.7(4)	C(24) – C(23) – C(22)	119.6(5)
N(2) – C(1) – C(14)	123.3(5)	C(7) – C(6) – C(5)	119.1(5)
N(13) – C(12) – C(22)	118.7(4)	C(42) – C(43) – C(44)	120.0(5)
N(11) – C(12) – N(13)	121.2(4)	C(43) – C(42) – C(41)	119.1(5)
N(11) – C(12) – C(22)	120.1(4)	C(9) – C(8) – C(7)	121.8(6)
N(13) – C(4) – C(5)	118.8(4)	C(17) – C(16) – C(15)	120.2(5)
N(3) – C(4) – N(13)	111.5(4)	C(8) – C(9) – C(10)	119.1(6)
N(3) – C(4) – C(5)	129.7(5)	C(42) – C(41) – C(40)	121.5(6)
C(27) – C(26) – C(25)	121.2(5)	C(42) – C(41) – H(41)	119.3
C(26) – C(25) – C(28)	122.6(4)	C(36) – C(35) – N(34)	120.8(6)
C(24) – C(25) – C(26)	117.5(4)	C(38) – C(35) – N(34)	118.4(6)
C(24) – C(25) – C(28)	119.9(5)	C(38) – C(35) – C(36)	120.8(6)
C(27) – C(22) – C(12)	119.0(5)	C(35) – C(36) – C(37)	119.8(9)
C(23) – C(22) – C(12)	121.1(5)	C(28) – C(29) – C(30)	121.6(5)
C(23) – C(22) – C(27)	119.9(4)	O(20) – C(21) – H(21A)	109.5
C(32) – C(31) – N(34)	120.4(5)	O(20) – C(21) – C(21B)	109.5
C(30) – C(31) – N(34)	121.2(5)	O(20) – C(21) – H(21C)	109.5
C(30) – C(31) – C(32)	118.4(5)	H(21A) – C(21) – H(21B)	109.5
C(33) – C(28) – C(25)	121.2(5)	H(21A) – C(21) – H(21C)	109.5
C(29) – C(28) – C(25)	121.5(5)	H(21B) – C(21) – H(21C)	109.5
C(29) – C(28) – C(33)	117.3(4)	C(2) – C(37) – C(36)	120.0(10)
C(26) – C(27) – C(22)	119.7(5)	C(35) – C(38) – C(3)	118.2(9)
C(27) – C(26) – C(25)	121.2(5)	C(37) – C(2) – C(3)	120.7(9)
C(14) – C(15) – C(16)	120.1(5)	C(2) – C(3) – C(38)	120.5(10)

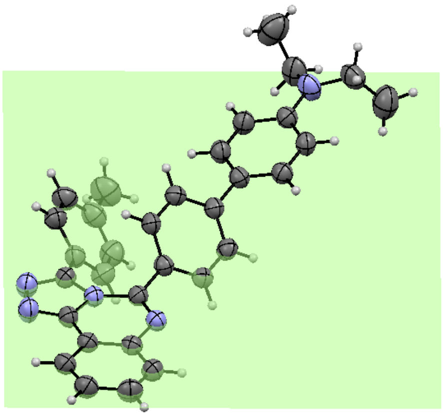

2a

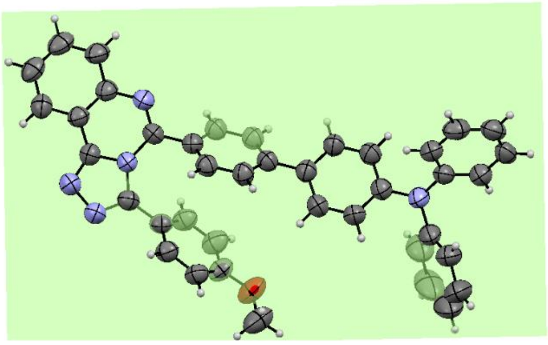

2e

Figure S10. Planarity of compounds **2a** and **2e**.

3. Absorption and emission spectra of fluorophores in toluene and MeCN

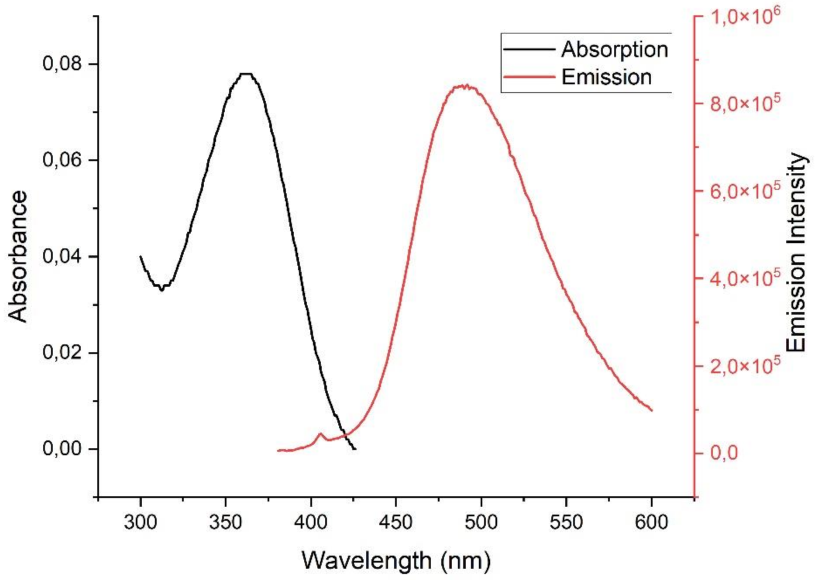

a

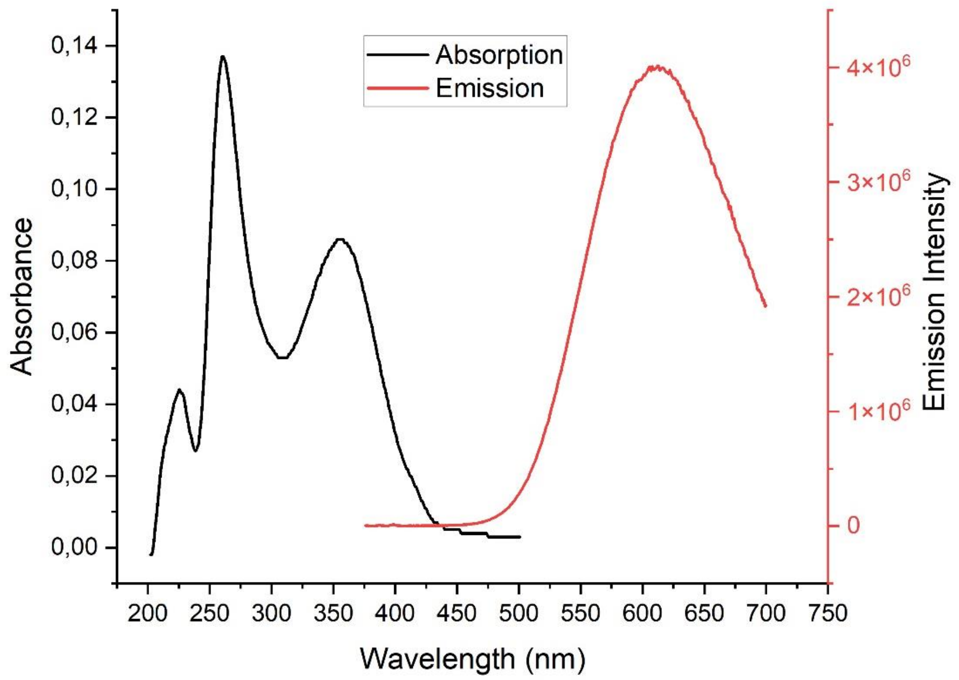

b

Figure S11. Absorption and emission spectra of compound **2a** in toluene (a) and in MeCN (b).

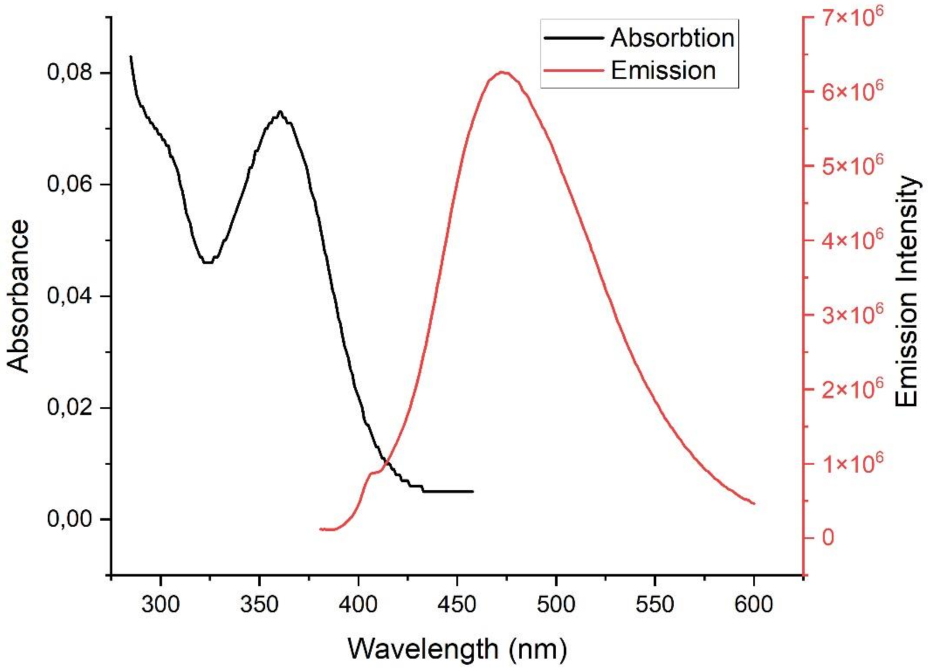

a

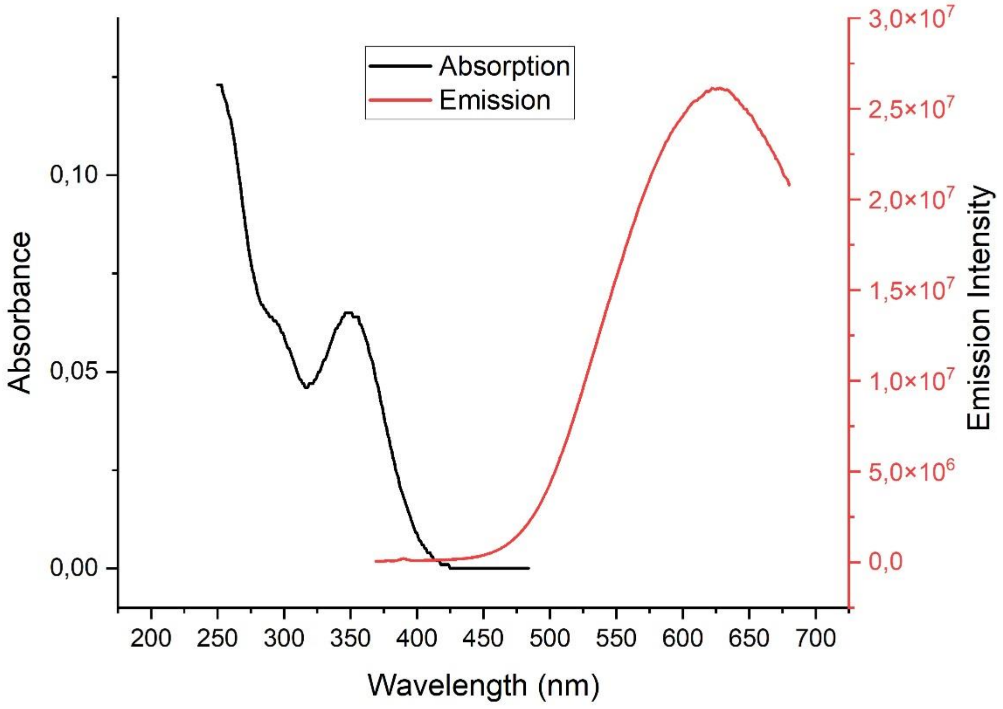

b

Figure S12. Absorption and emission spectra of compound **2b** in toluene (a) and in MeCN (b).

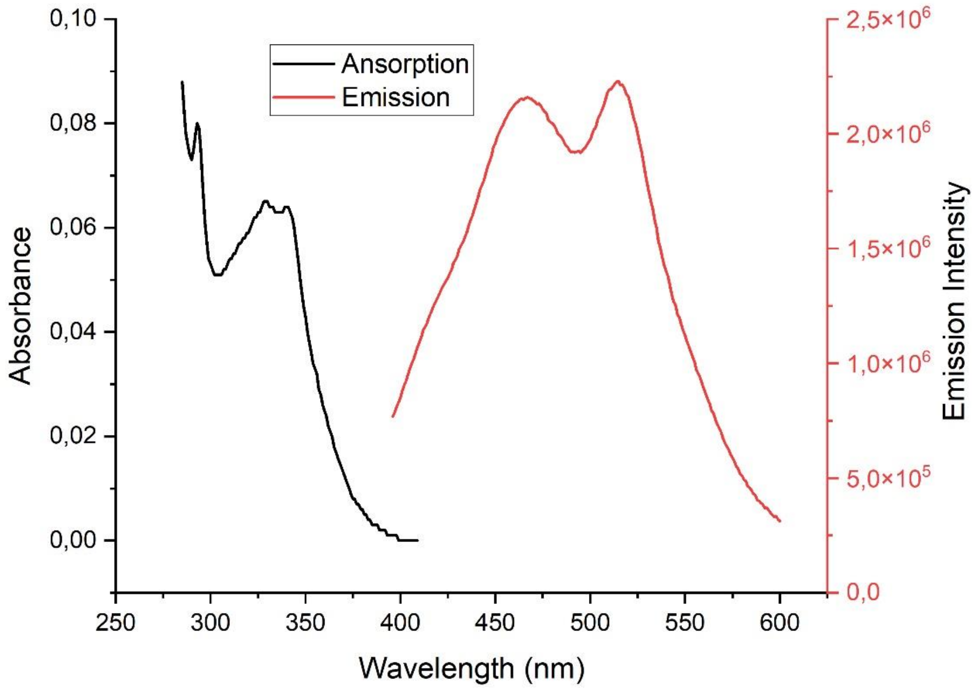

a

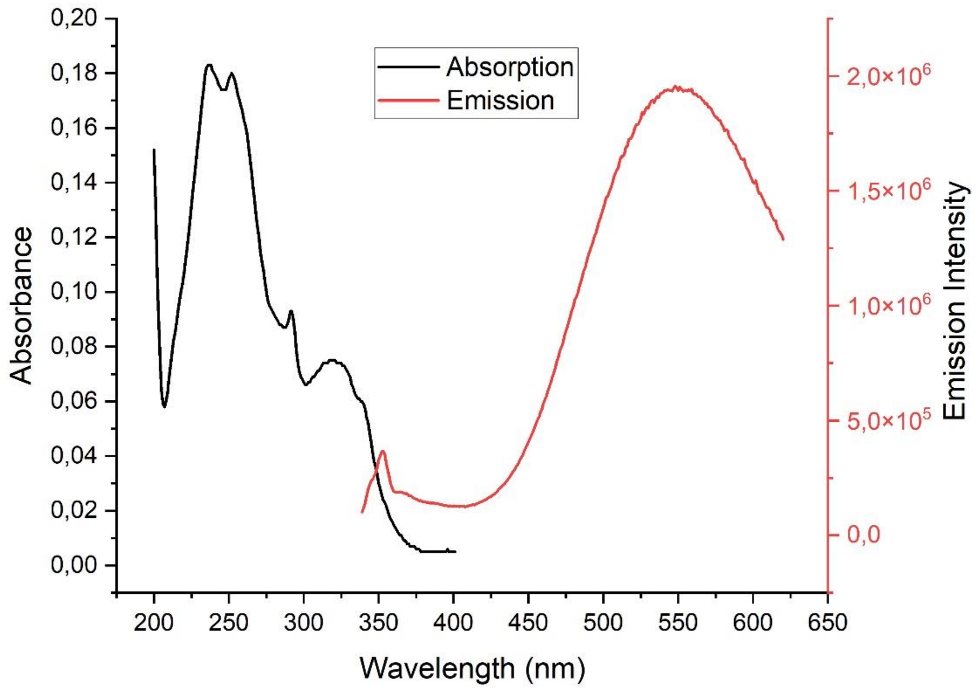

b

Figure S13. Absorption and emission spectra of compound **2c** in toluene (a) and in MeCN (b).

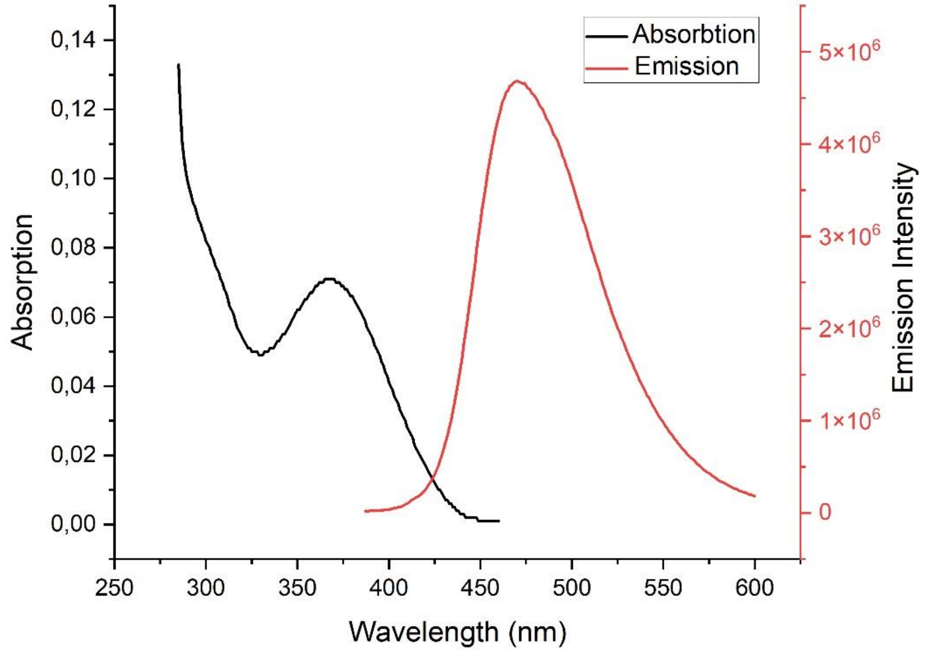

a

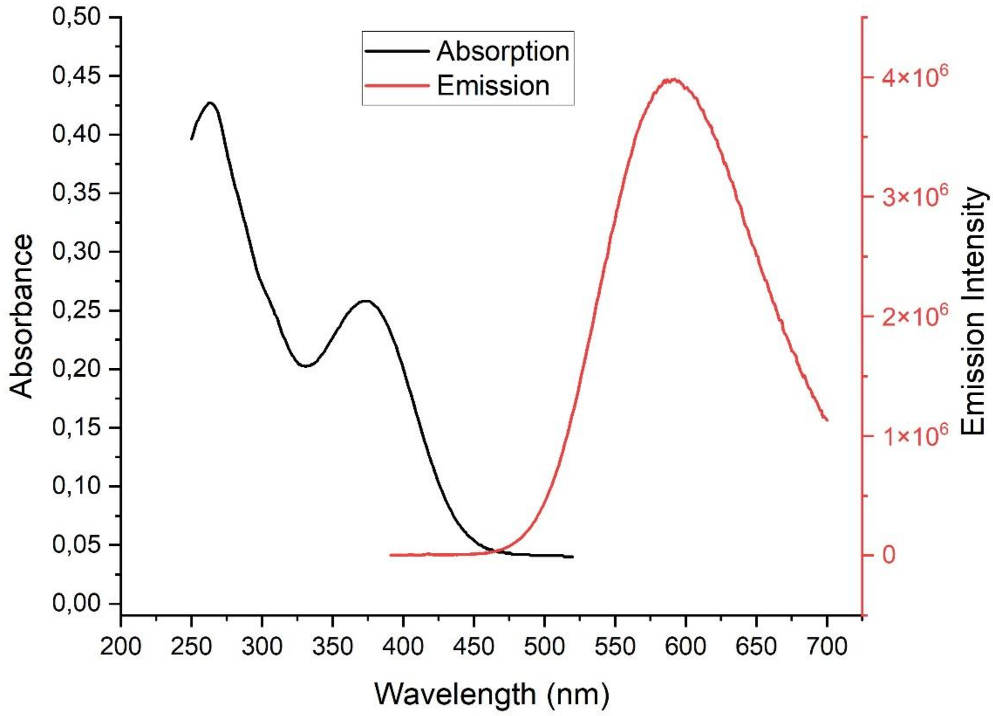

b

Figure S14. Absorption and emission spectra of compound **2d** in toluene (a) and in MeCN (b).

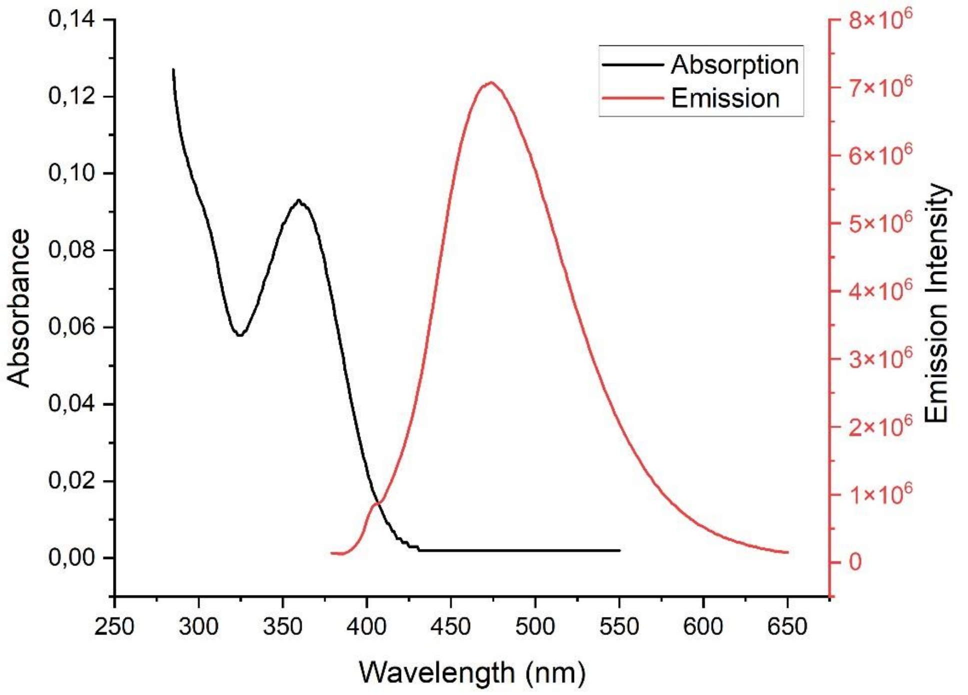

a

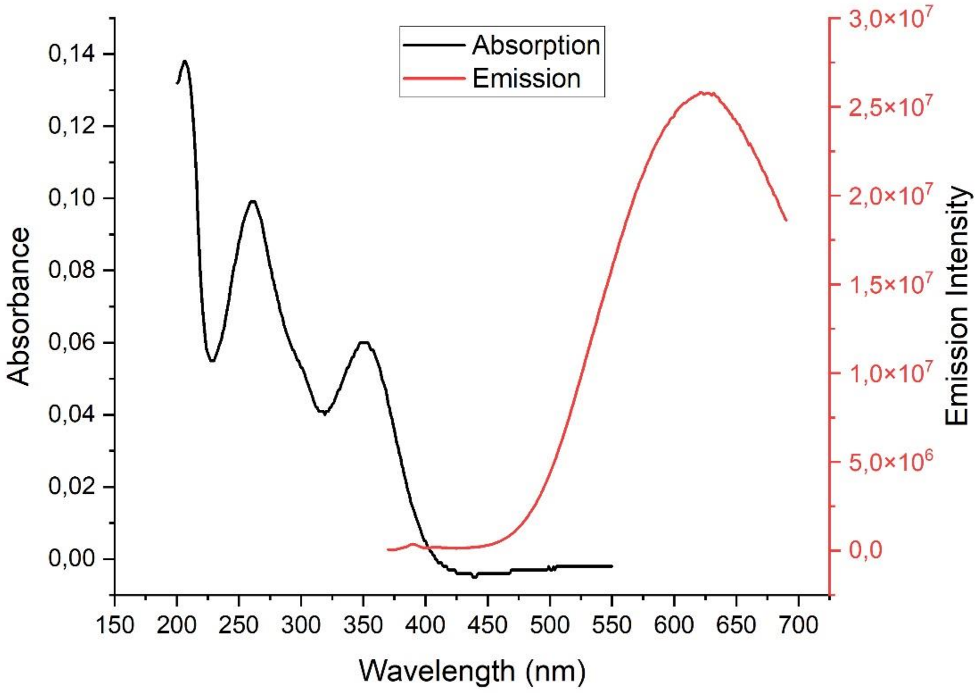

b

Figure S15. Absorption and emission spectra of compound **2e** in toluene (a) and in MeCN (b).

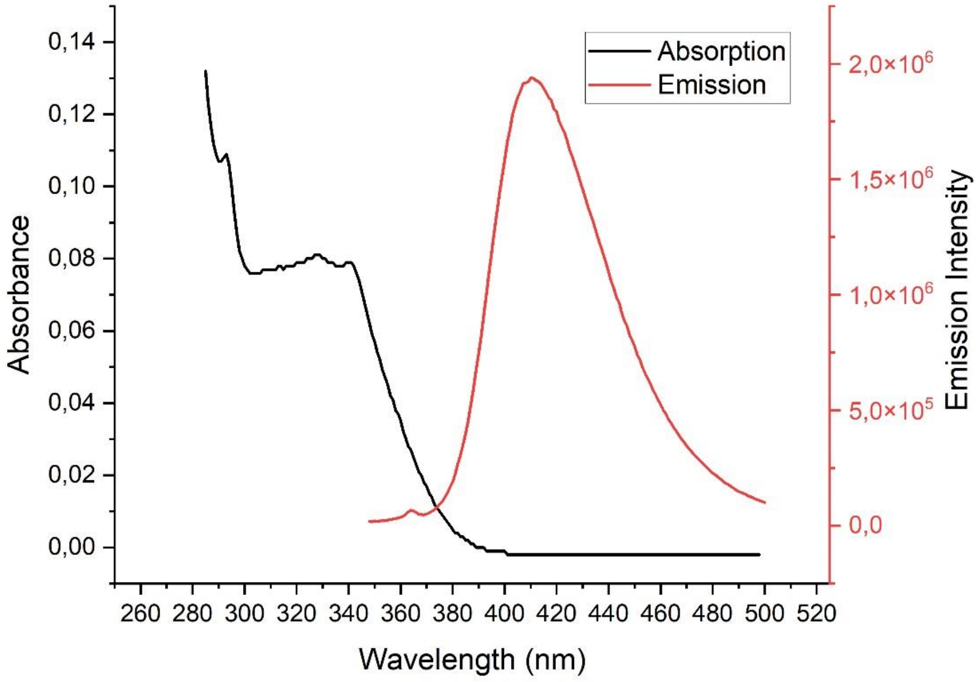

a

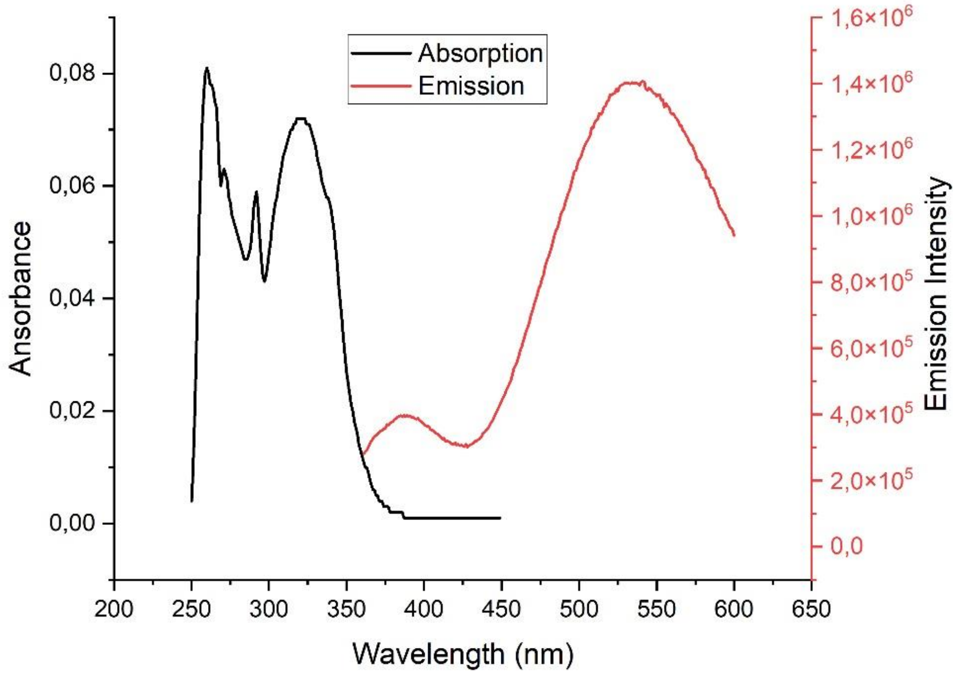

b

Figure S16. Absorption and emission spectra of compound **2f** in toluene (a) and in MeCN (b).

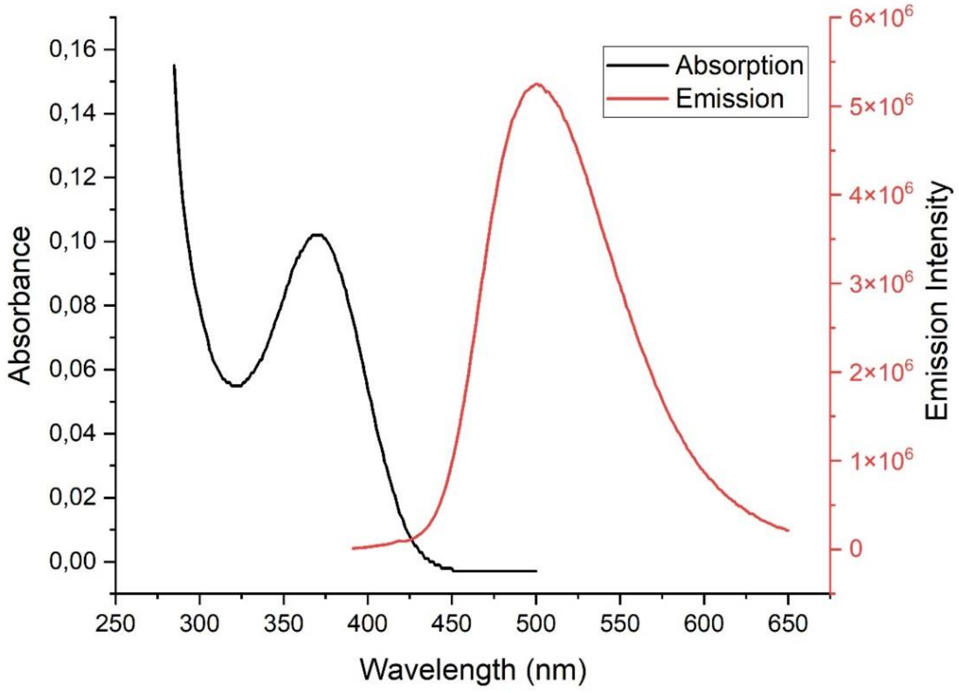

a

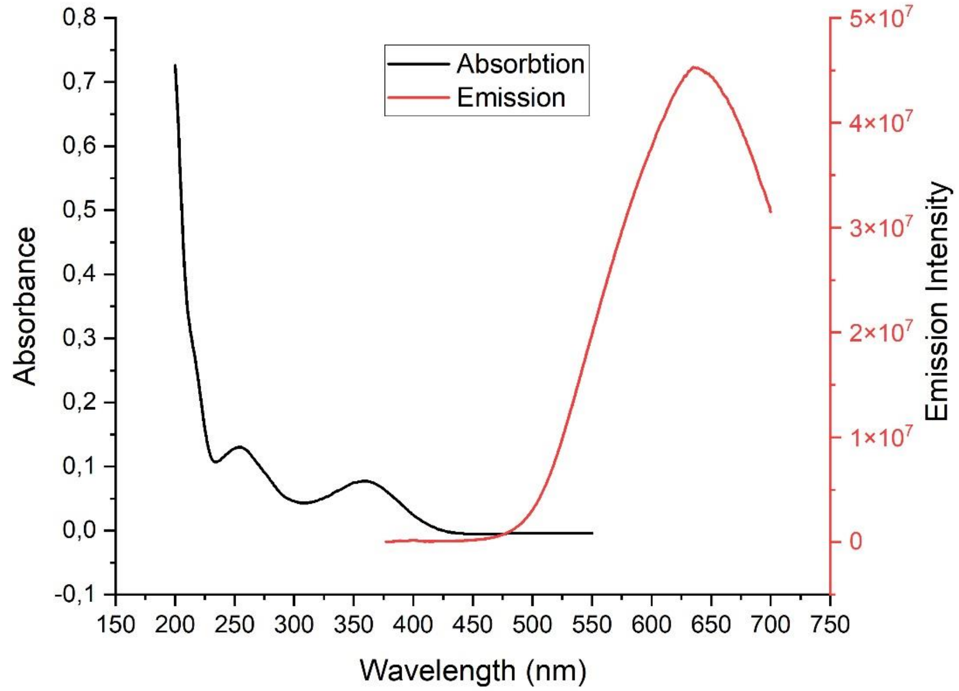

b

Figure S17. Absorption and emission spectra of compound **2g** in toluene (a) and in MeCN (b).

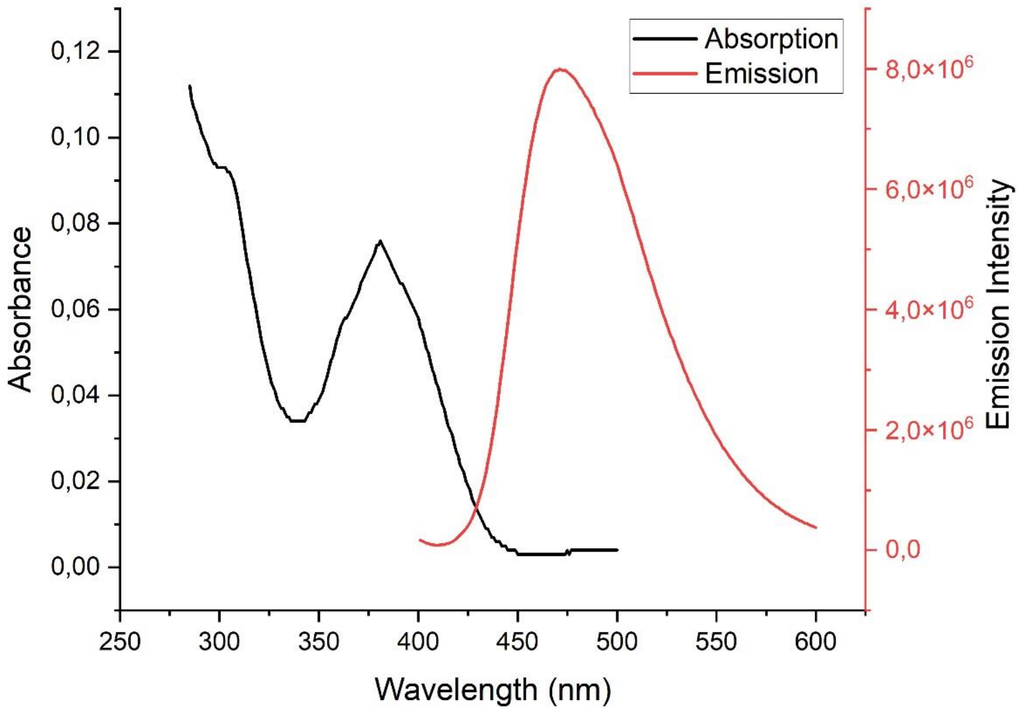

a

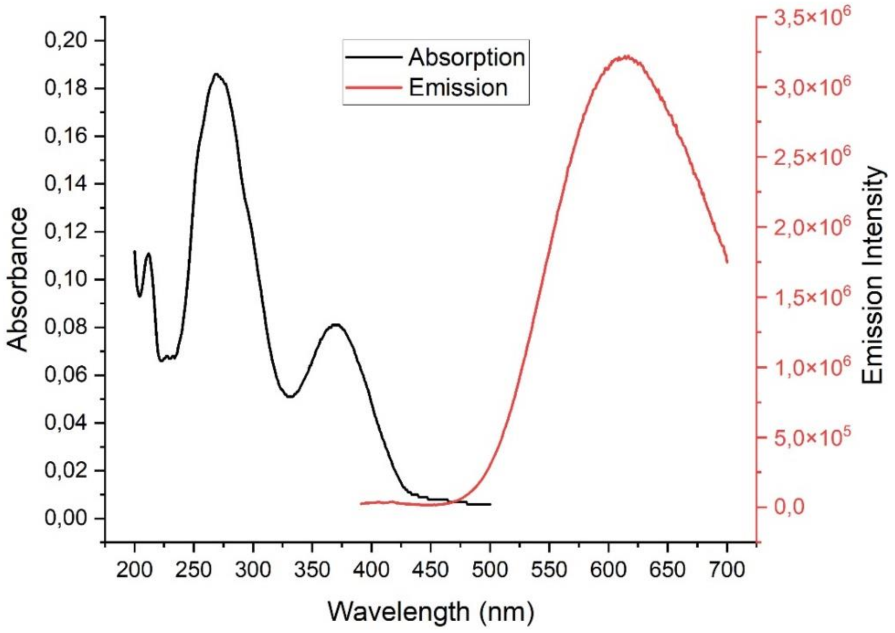

b

Figure S18. Absorption and emission spectra of compound **2h** in toluene (a) and in MeCN (b).

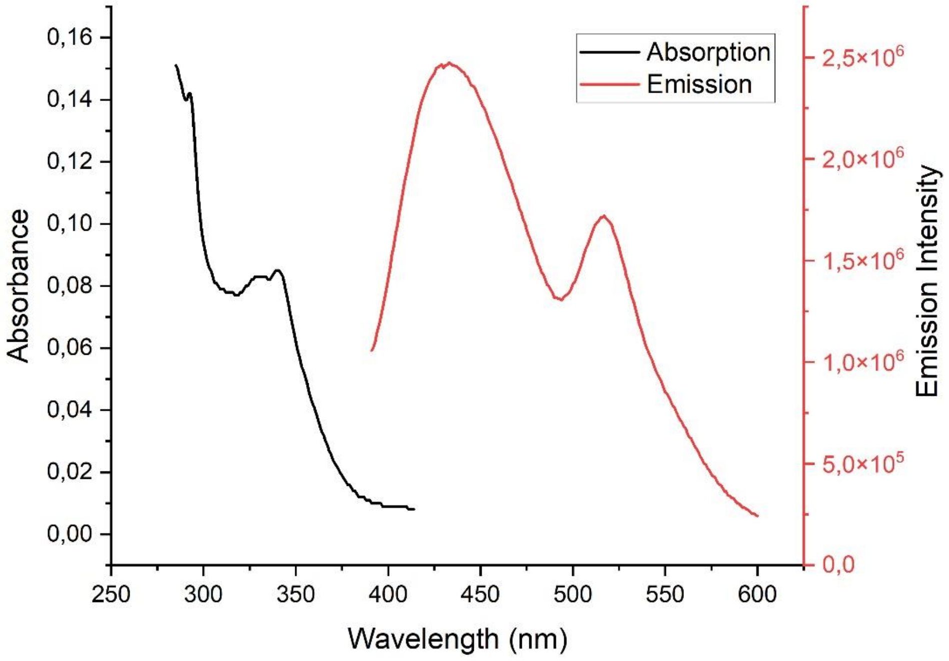

a

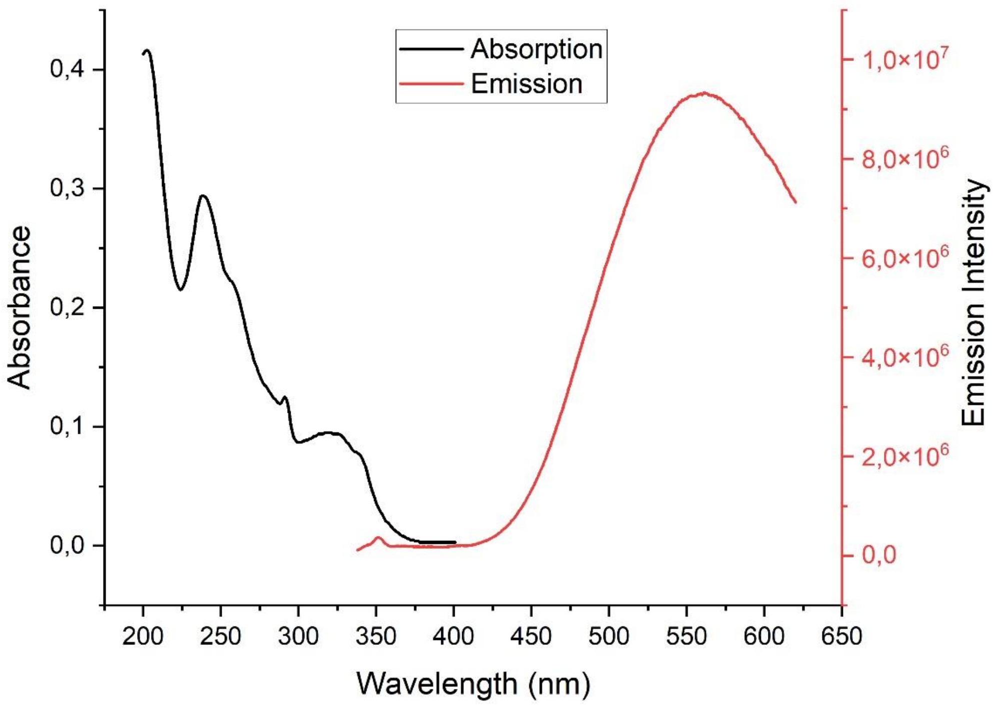

b

Figure S19. Absorption and emission spectra of compound **2i** in toluene (a) and in MeCN (b).

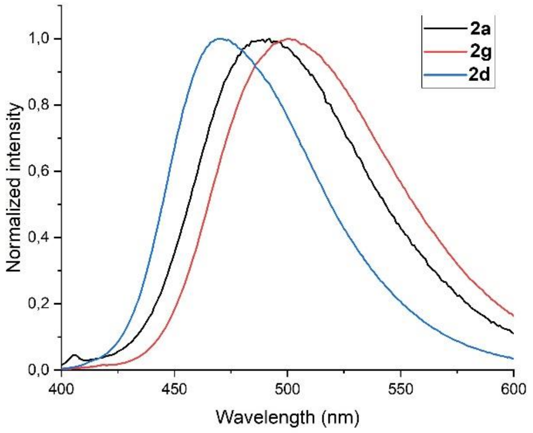

a

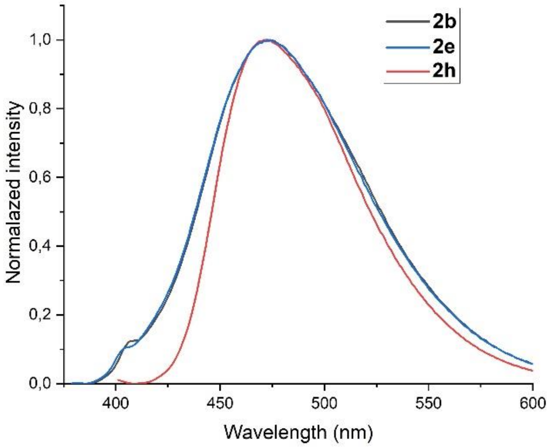

b

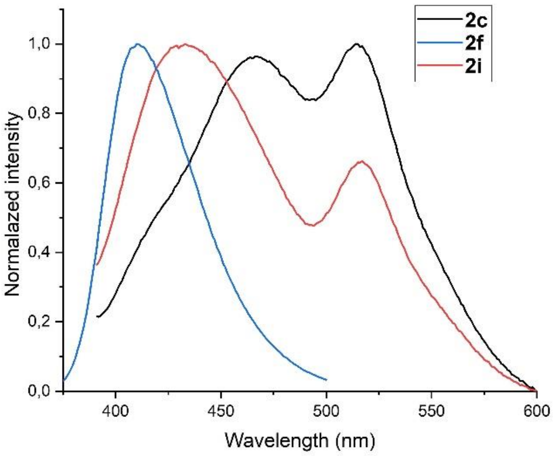

Figure S20. Emission spectra of compounds **2a, 2d, 2g** (a) **2b, 2e, 2h** (b) and **2c, 2f, 2i** (c) in toluene.

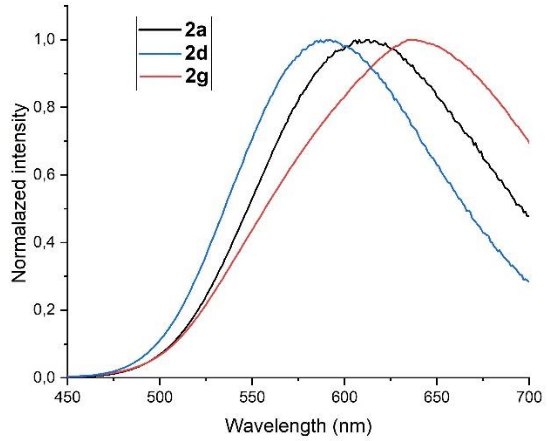

a

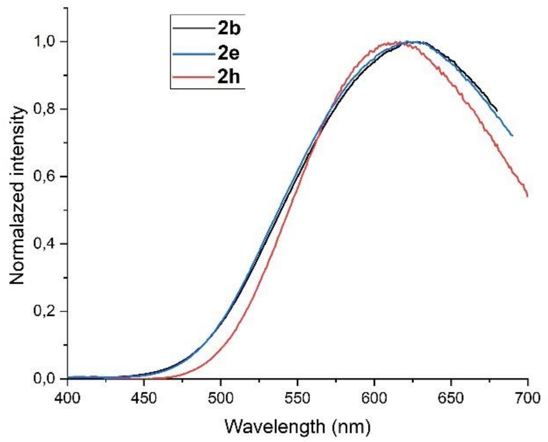

b

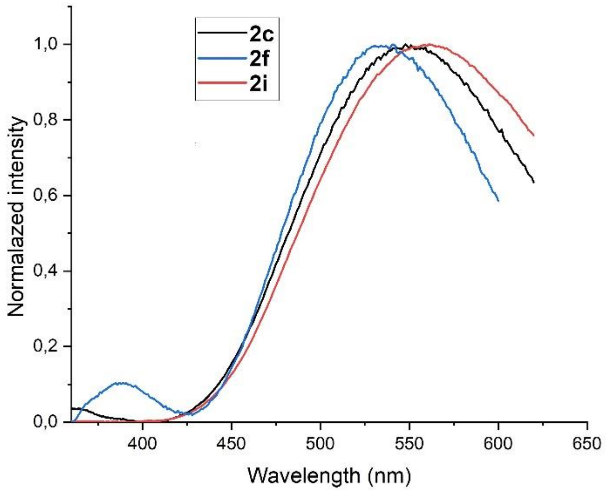

Figure S21. Emission spectra of compounds **2a, 2d, 2g** (a) **2b, 2e, 2h** (b) and **2c, 2f, 2i** (c) in MeCN.

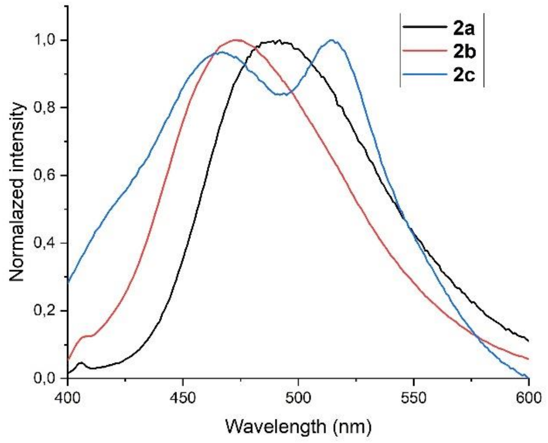

a

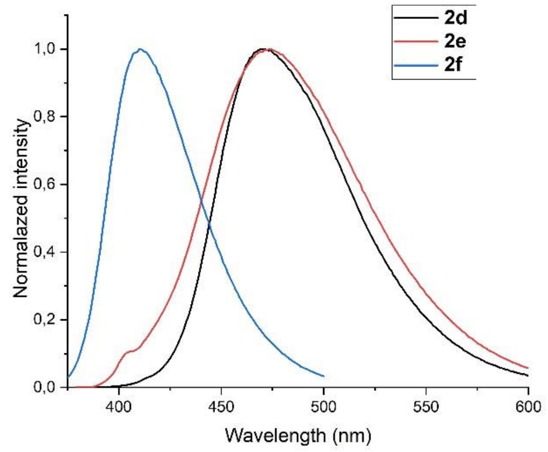

b

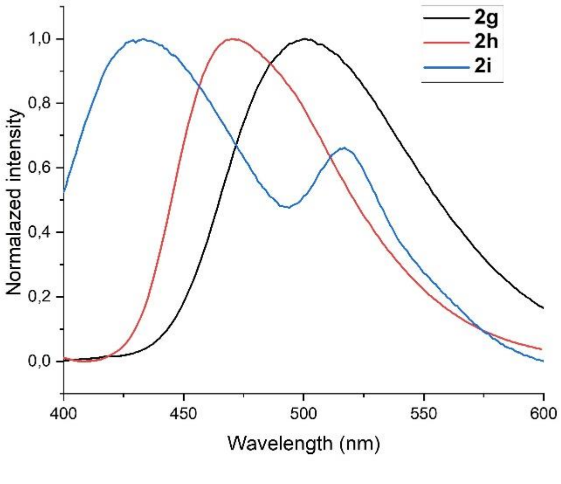

c

Figure S22. Emission spectra of compounds **2a-c** (a) **2d-f** (b) and **2g-i** (c) in toluene.

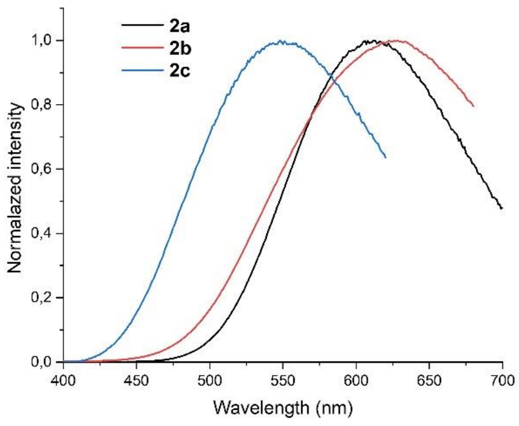

a

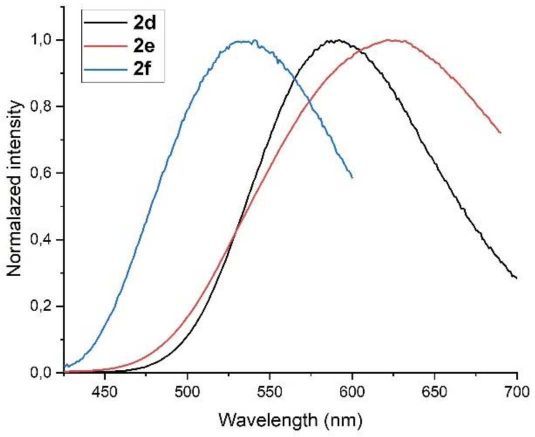

b

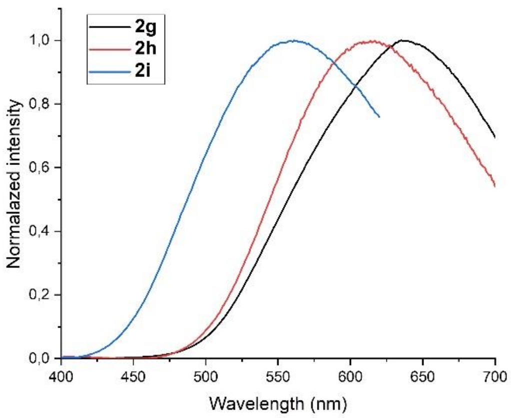

c

Figure S23. Emission spectra of compounds **2a-c** (a) **2d-f** (b) and **2g-i** (c) in MeCN.

4. Emission spectra of fluorophores in solid state

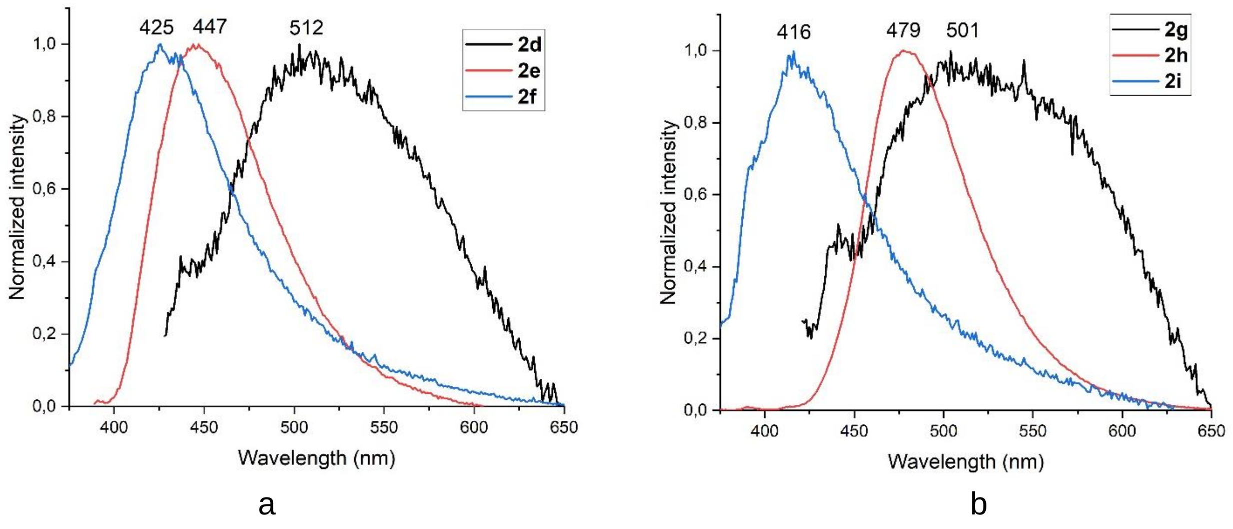

Figure S24. The emission spectra of compounds **2d-f** (a) and **2g-i** (b) in solid state.

5. Solvatochromic study for compounds 2

Table S5. Orientation polarizability for solvents (Δf), absorption and emission maxima (λ_{abs}, λ_{em}, nm) and Stokes shift (nm, cm^{-1}) of **2a** in different solvents.

Solvent	Δf	λ_{abs}, nm	λ_{em}, nm	Stokes shift, nm	Stokes shift, cm^{-1}
Cyclohexane	0.001	353	451	98	6156
Toluene	0.0126	361	491	130	7334
THF	0.21	360	553	193	9695
DCM	0.22	364	555	191	9455
DMSO	0.26	360	632	272	11955
MeCN	0.3	356	612	256	11750
MeOH	0.31	360	648	288	12346

Table S6. Orientation polarizability for solvents (Δf), absorption and emission maxima (λ_{abs}, λ_{em}, nm) and Stokes shift (nm, cm^{-1}) of **2d** in different solvents.

Solvent	Δf	λ_{abs}, nm	λ_{em}, nm	Stokes shift, nm	Stokes shift, cm^{-1}
Cyclohexane	0.001	373	**424**, 452	71	3225
Toluene	0.0126	367	472	105	6062
THF	0.21	381	538	157	7659
DCM	0.22	382	531	149	7346
DMSO	0.26	384	616	232	9808
MeCN	0.3	371	592	221	10062
MeOH	0.31	375	614	239	10380

Table S7. Orientation polarizability for solvents (Δf), absorption and emission maxima (λ_{abs}, λ_{em}, nm) and Stokes shift (nm, cm^{-1}) of **2g** in different solvents.

Solvent	Δf	λ_{abs}, nm	λem, nm	Stokes shift, nm	Stokes shift, cm^{-1}
Cyclohexane	0,001	359	460	101	6116
Toluene	0,0126	371	502	131	7034
THF	0,21	366	565	199	9623
DCM	0,22	367	565	198	9549
DMSO	0,26	364	643	279	11920
MeCN	0,3	357	640	283	12386
MeOH	0,31	364	651	287	12112

Table S8. Orientation polarizability for solvents (Δf), absorption and emission maxima (λ_{abs}, λ_{em}, nm) and Stokes shift (nm, cm^{-1}) of **2h** in different solvents.

Solvent	Δf	λ_{abs}, nm	λ_{em}, nm	Stokes shift, nm	Stokes shift, cm^{-1}
Cyclohexane	0.001	380	427, **468**	88	4948
Toluene	0.0126	381	473	92	5105
THF	0.21	378	546	168	8140
DCM	0.22	378	554	176	8404
DMSO	0.26	378	624	246	10429
MeCN	0.3	371	615	244	10694
MeOH	0.31	373	624	251	10784

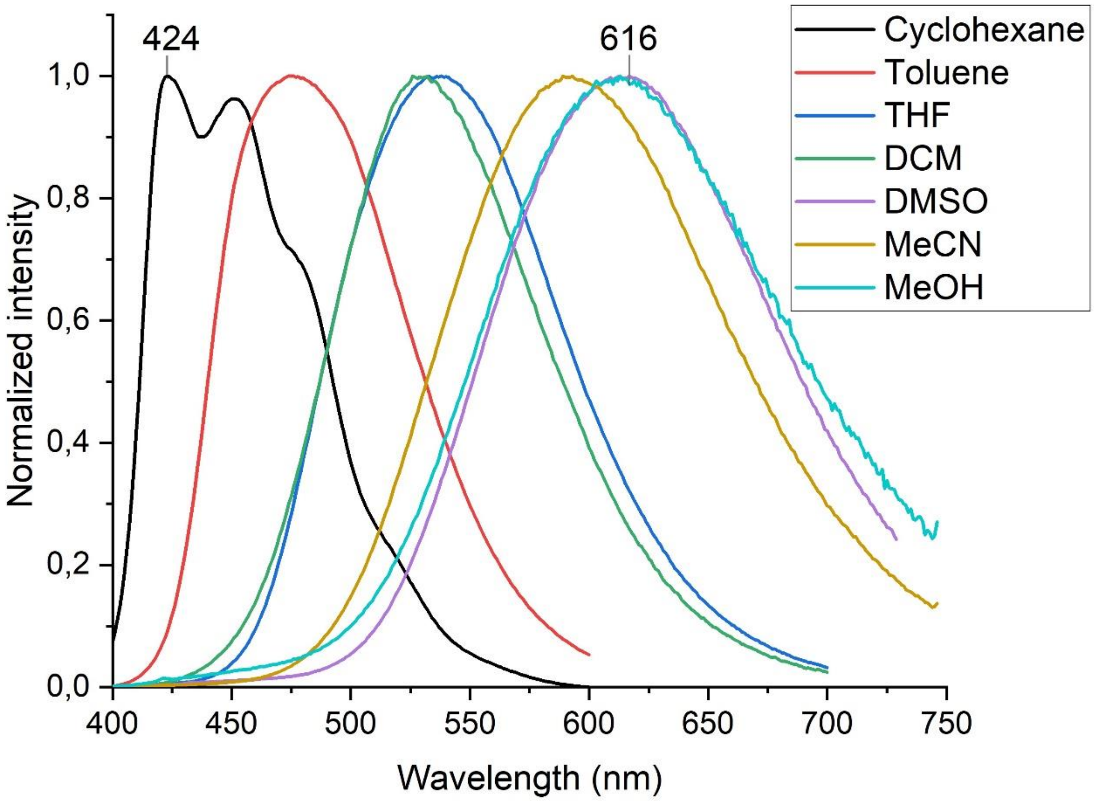

424
616
Cyclohexane
Toluene
THF
DCM
DMSO
MeCN
MeOH
Normalized intensity
1,0
0,8
0,6
0,4
0,2
0,0
400
450
500
550
600
650
700
750
Wavelength (nm)

a

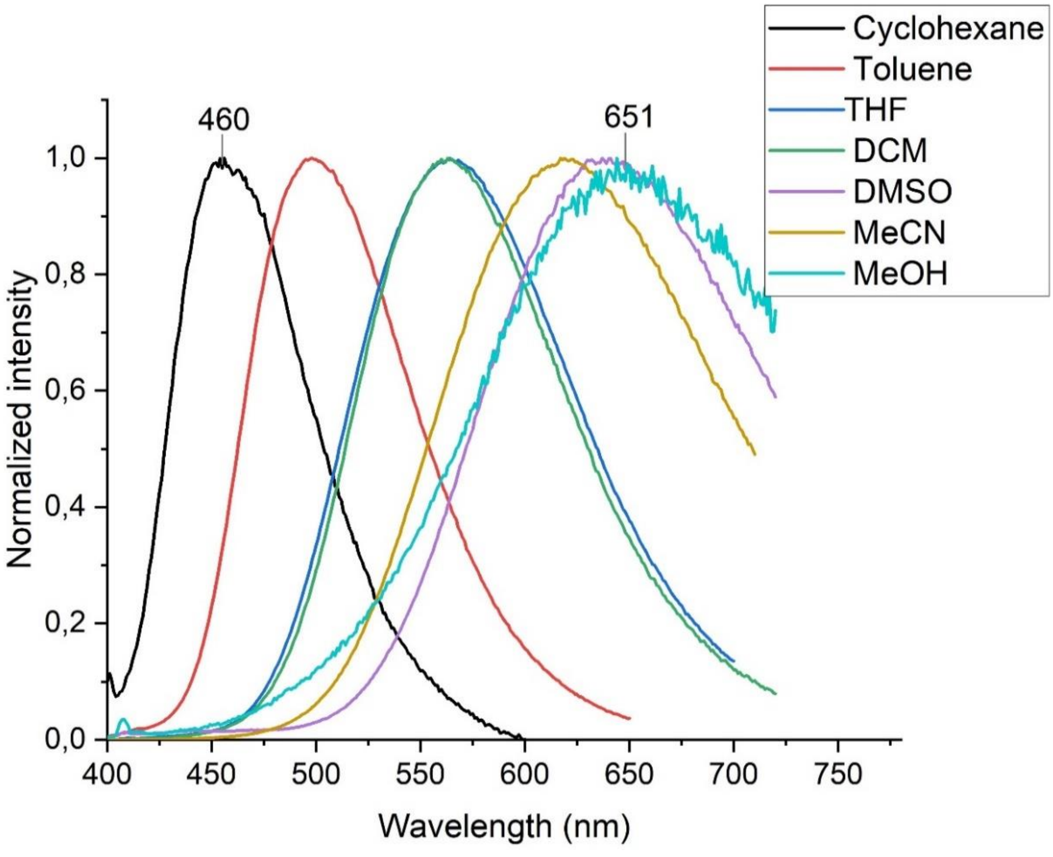

460
651
Cyclohexane
Toluene
THF
DCM
DMSO
MeCN
MeOH
Normalized intensity
1,0
0,8
0,6
0,4
0,2
0,0
400
450
500
550
600
650
700
750
Wavelength (nm)

b

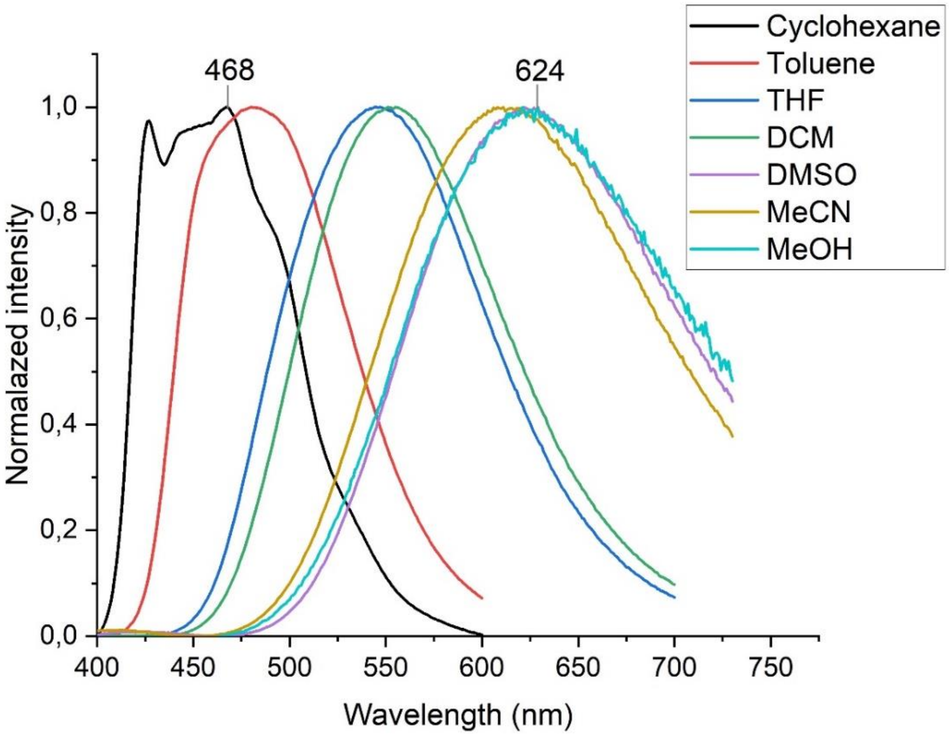

c

Figure S25. Fluorescence spectra of compound **2d** (a), **2g** (b) and **2h** (c) in different solvents.

6. Absorption and emission behavior of compounds 2 in MeCN/water mixture

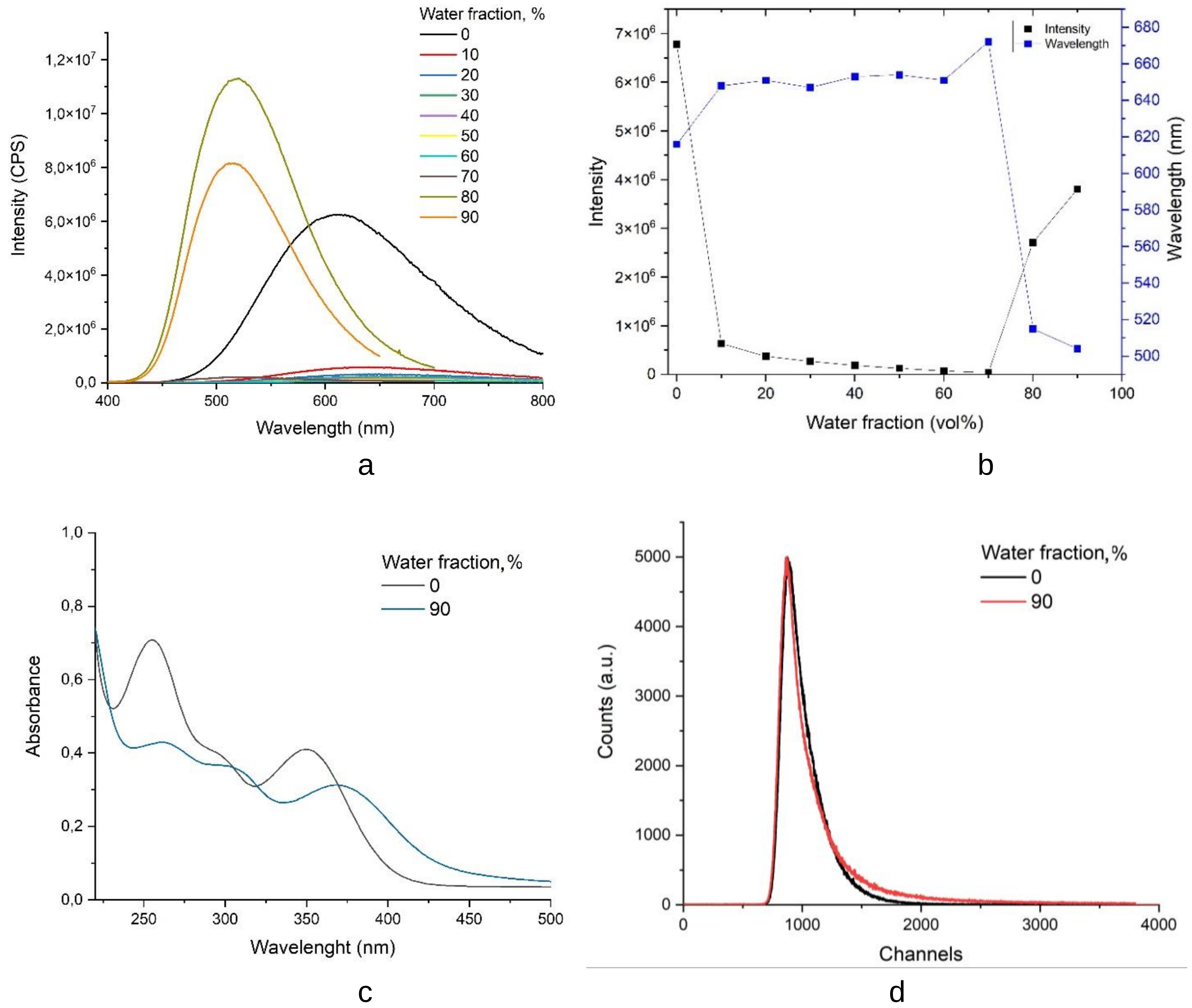

Figure S26. (a) The fluorescence spectra of **2b** in MeCN and MeCN/water mixtures with different water fractions (f_W); (b) a plot of relative PL intensity (I/I_0) and wavelength at emission maxima of **2b** versus the composition of the water fraction (vol %); (c) The absorption spectra of **2b** in MeCN and MeCN/water mixture (90%); (d) Fluorescence decay curves of **2b** in pure MeCN and MeCN/water mixture.T=23 ºC.

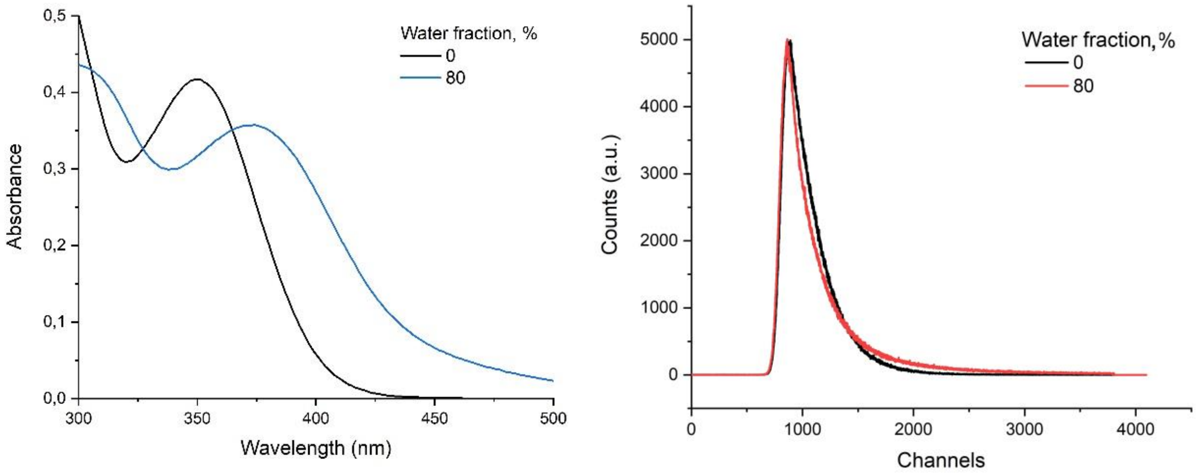

a b

Figure S27. (a) The absorption spectra of **2e** in MeCN and MeCN/water mixture (90%); (b) Fluorescence decay curves of **2e** in pure MeCN and MeCN/water mixture.T=23 ºC.

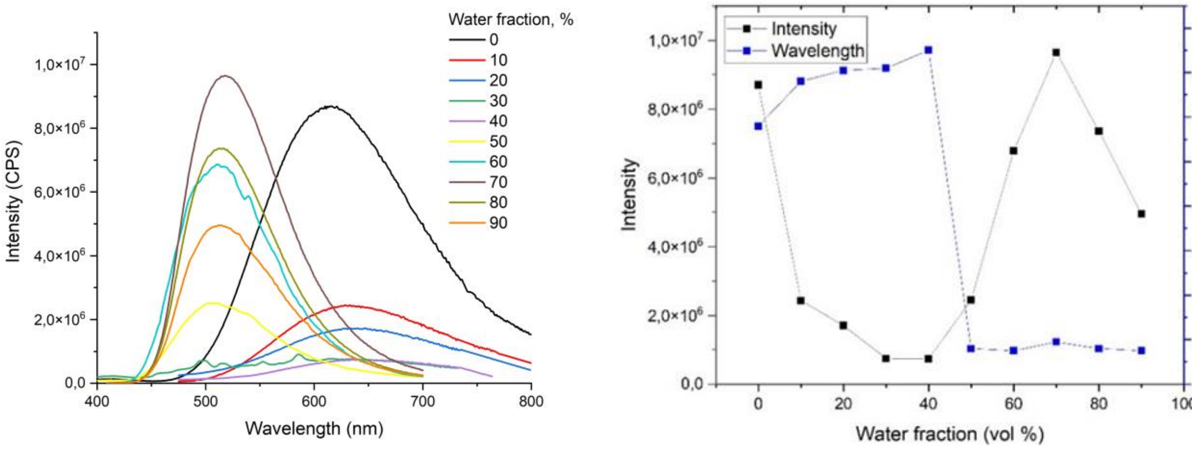

a b

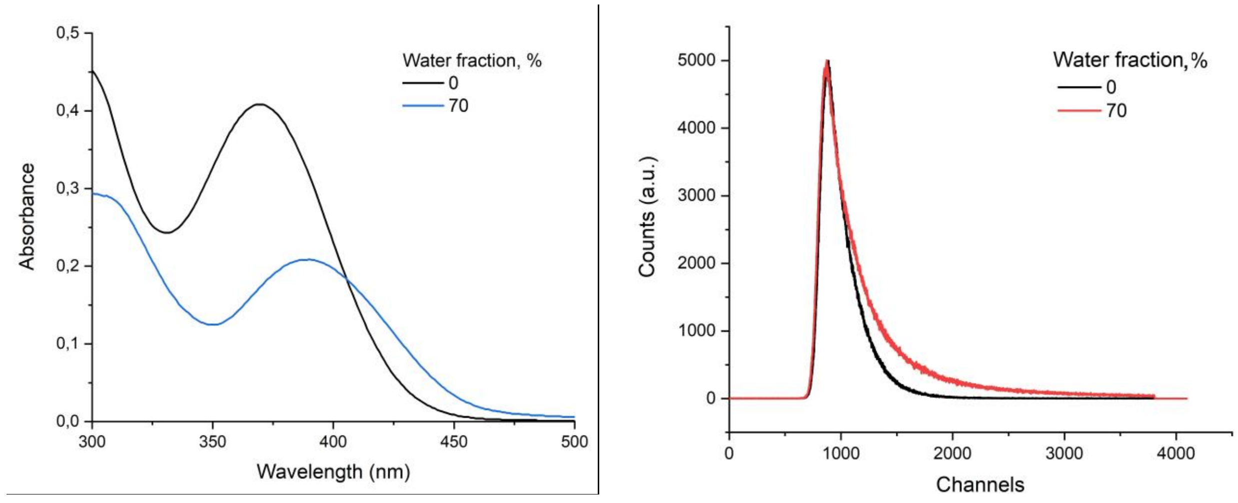

c d

Figure S28. (a) The fluorescence spectra of **2h** in MeCN and MeCN/water mixtures with different water fractions (f_W); (b) a plot of relative PL intensity (I/I_0) and wavelength at emission maxima of **2h** versus the composition of the water fraction (vol %); (c) The

absorption spectra of **2h** in MeCN and MeCN/water mixture (90%); (d) Fluorescence decay curves of **2h** in pure MeCN and MeCN/water mixture. T = 23 ºC.

Table S9. Detailed data of the fluorescence lifetime measurements of **2b, 2e, 2h**.

Comp.	Solvent	$τ_1$, ns[a]	$α_1$[b]	$τ_2$, ns[a]	$α_2$[b]	$τ_3$, ns[a]	$α_3$[b]	$τ_{av}$, ns[c]	$χ^2$[d]
**2b**	MeCN	2.67	7.35	2.36	92.65			**2.38**	1.1003
	MeCN/water (1:9)	6.44	25.37	**2.96**	**58.82**	1.27	15.81	**3.57**	1.0649
**2e**	MeCN	3.18	1					**3.17**	1.1831
	MeCN/water (2:8)	**7.76**	**98.99**	5.36	0.60	1.51	0.41	**7.72**	1.2621
**2h**	MeCN	2.46	1					**2.46**	1.3224
	MeCN/water (3:7)	6.21	0.26	1.73	0.15	**1.35**	**99.59**	**1.36**	1.2688

[a]Decay time, [b]Fractional contribution, [c]Weighted average decay time $τ_{av} = Σ (τ_i × α_i)$, [d]Quality of fitting

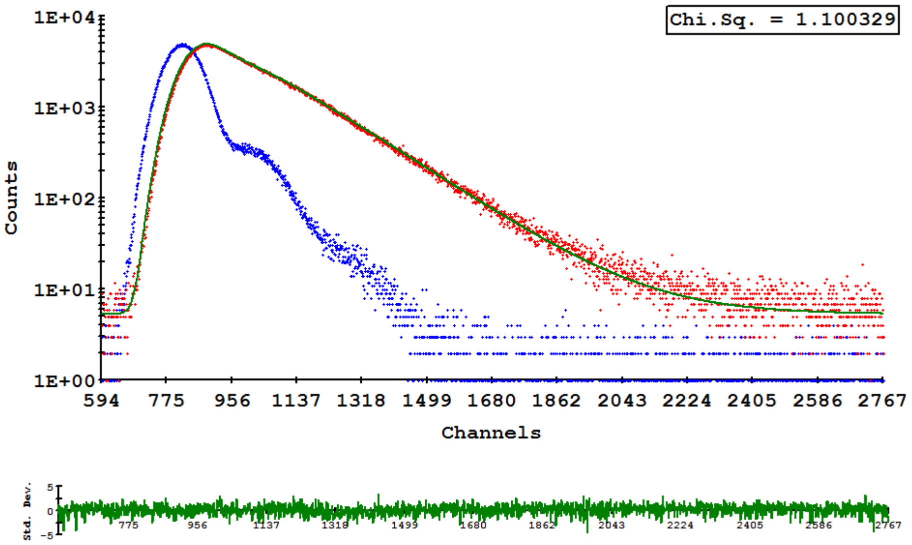

a

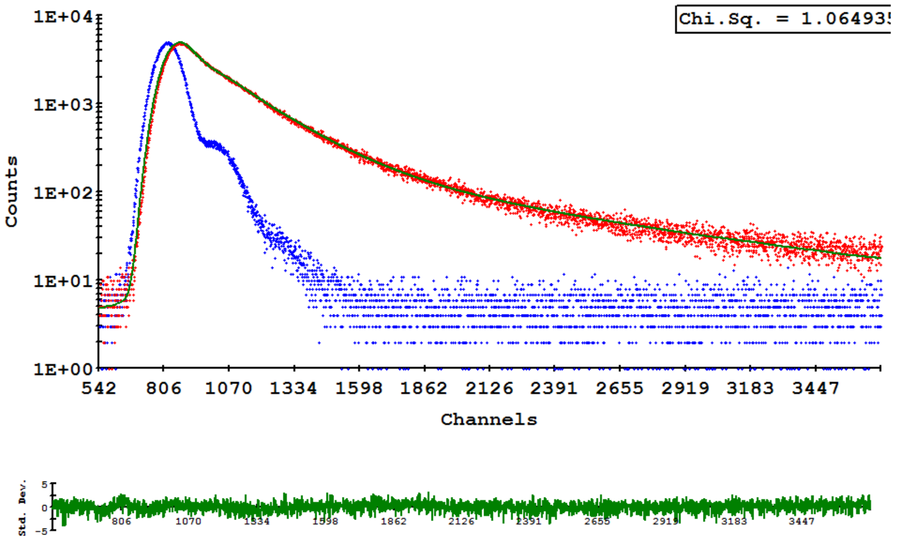

b

Figure S29. Time-resolved fluorescence lifetime decay profile of **2b** in MeCN, $λ_{em}$ = 627 nm (a) and in MeCN/water (90%), $λ_{em}$ = 500 nm (b), instrumental response function (IRF, blue). $λ_{ex}$ = 369 nm.

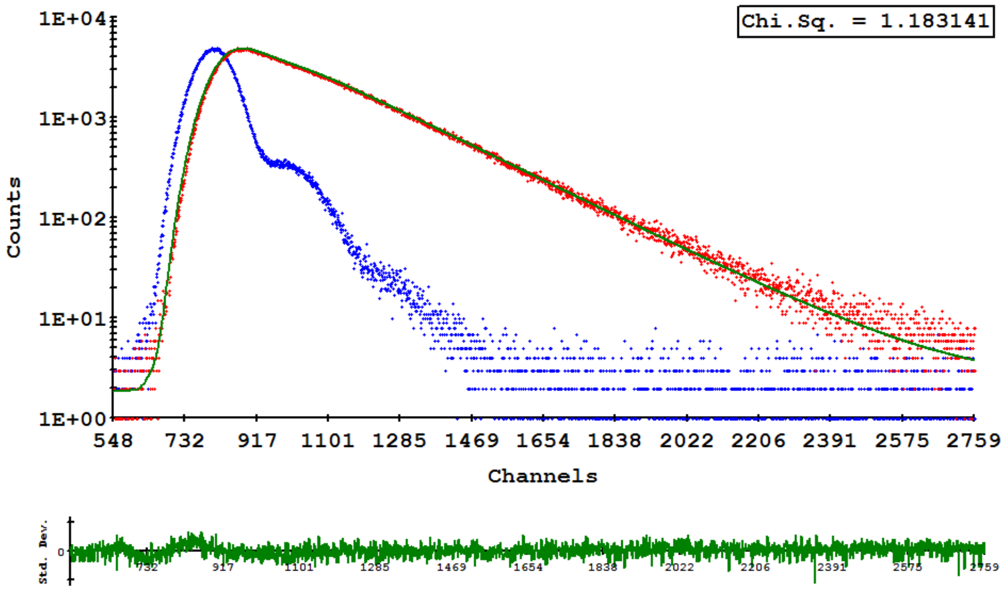

a

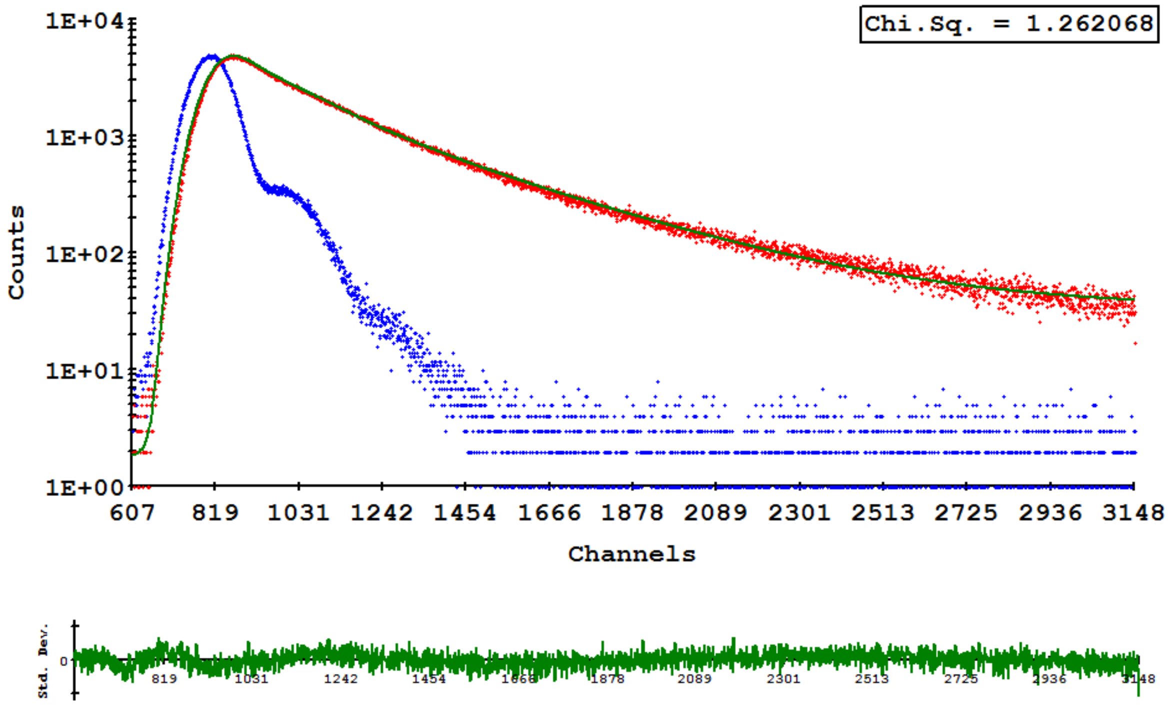

b

Figure S30. Time-resolved fluorescence lifetime decay profile of **2e** in MeCN, λ_{em} = 625 nm (a) and in MeCN/water (80%), λ_{em} = 521 nm (b), instrumental response function (IRF, blue). λ_{ex} = 369 nm.

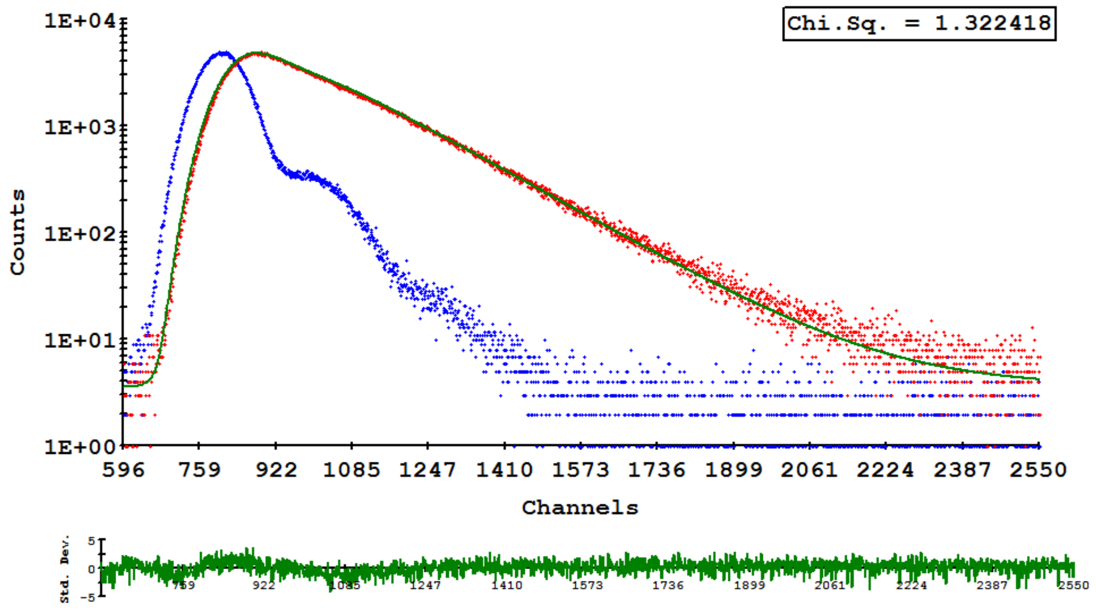

a

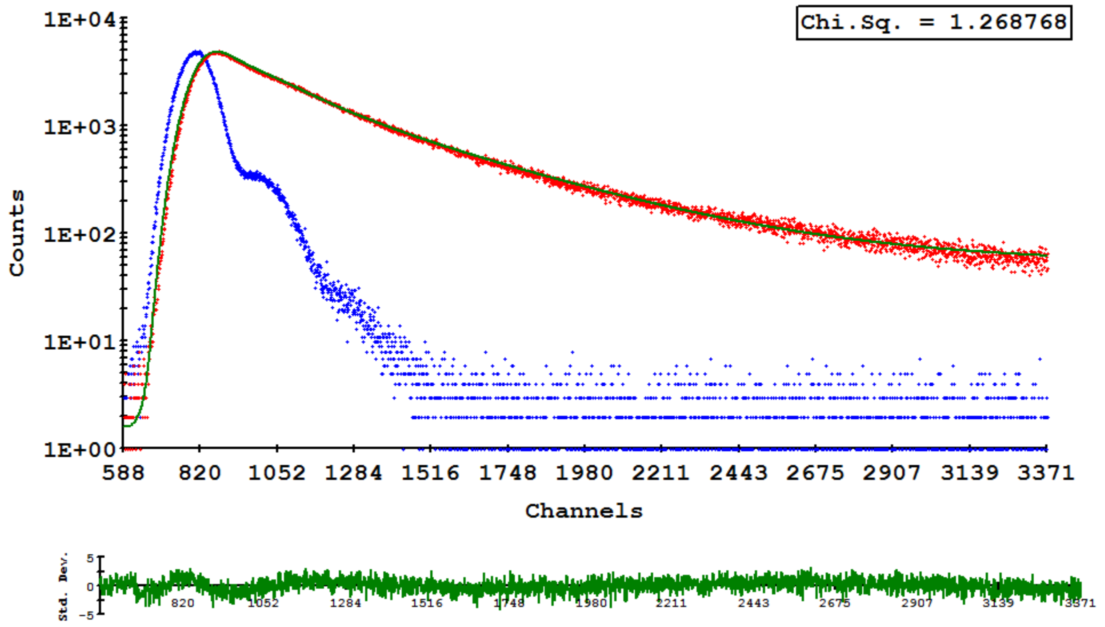

b

Figure S31. Time-resolved fluorescence lifetime decay profile of **2h** in MeCN, λ_{em} = 615 nm (a) and in MeCN/water (70%), λ_{em} = 518 nm (b), instrumental response function (IRF, blue). λ_{ex} = 369 nm.

7. Absorption and fluorescence behavior of compounds 2e and 2h in acidic media

	**2e**	**2h**
Daylight	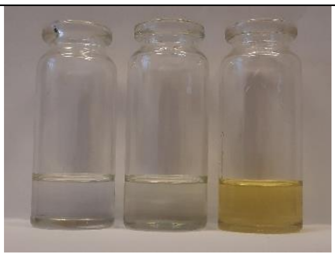<br>1 2 3<br>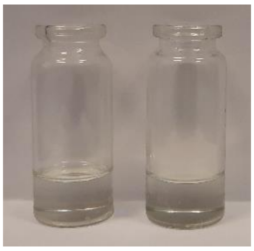<br>1 4	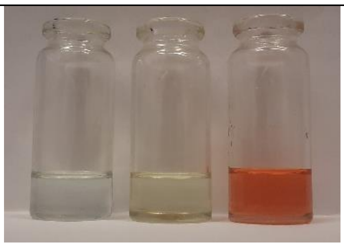<br>1 2 3<br>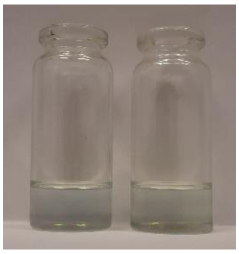<br>1 4
UV-light	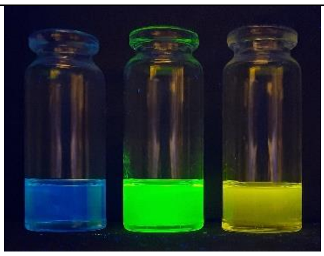<br>1 2 3<br>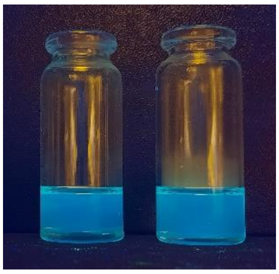<br>1 4	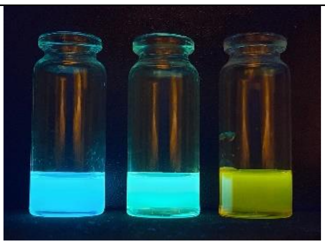<br>1 2 3<br>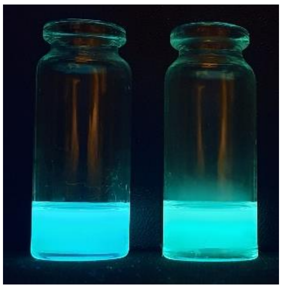<br>1 4

Figure S32. Photos of **2e** and **2h** in toluene before (1), after addition of TFA (2), after addition of excess of TFA (3) and after subsequent addition of TFA and then TEA (4) under UV-light and daylight, respectively.

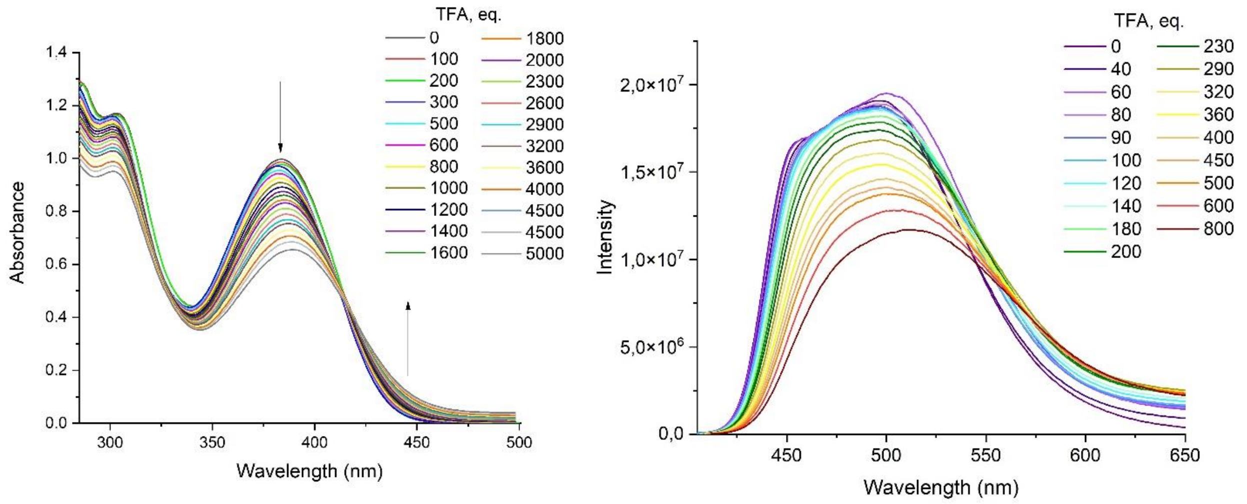

a b

Figure S33. Changes in absorption (a) and emission (b) spectra of the toluene solution (c = 10^{-5} M) of **2h** upon gradual addition of TFA.
